# *Hnf1b* haploinsufficiency differentially affects developmental target genes in a new renal cysts and diabetes mouse model

**DOI:** 10.1242/dmm.047498

**Published:** 2021-05-04

**Authors:** Leticia L. Niborski, Mélanie Paces-Fessy, Pierbruno Ricci, Adeline Bourgeois, Pedro Magalhães, Maria Kuzma-Kuzniarska, Celine Lesaulnier, Martin Reczko, Edwige Declercq, Petra Zürbig, Alain Doucet, Muriel Umbhauer, Silvia Cereghini

**Affiliations:** 1 Sorbonne Université, CNRS, Institut de Biologie Paris Seine, Laboratoire de Biologie du Développement, IBPS, UMR7622, F-75005 Paris, France; 2 Mosaiques Diagnostics, 30659 Hannover, Germany; 3 Department of Pediatric Nephrology, Hannover Medical School, 30625 Hannover, Germany; 4 Biomedical Sciences Research Center Alexander Fleming, Institute for Fundamental Biomedical Science, 16672 Athens, Greece; 5 Sorbonne Université, Université Paris Descartes, UMRS 1138, CNRS, ERL 8228, Centre de Recherche des Cordeliers, F-75006 Paris, France

**Keywords:** HNF1B transcription factor, RCAD syndrome, Gene dosage, Glomerular and proximal tubule cysts, Mouse models, Transcriptomics

## Abstract

Heterozygous mutations in *HNF1B* cause the complex syndrome renal cysts and diabetes (RCAD), characterized by developmental abnormalities of the kidneys, genital tracts and pancreas, and a variety of renal, pancreas and liver dysfunctions. The pathogenesis underlying this syndrome remains unclear as mice with heterozygous null mutations have no phenotype, while constitutive/conditional *Hnf1b* ablation leads to more severe phenotypes. We generated a novel mouse model carrying an identified human mutation at the intron-2 splice donor site. Unlike heterozygous mice previously characterized, mice heterozygous for the splicing mutation exhibited decreased HNF1B protein levels and bilateral renal cysts from embryonic day 15, originated from glomeruli, early proximal tubules (PTs) and intermediate nephron segments, concurrently with delayed PT differentiation, hydronephrosis and rare genital tract anomalies. Consistently, mRNA sequencing showed that most downregulated genes in embryonic kidneys were primarily expressed in early PTs and the loop of Henle and involved in ion/drug transport, organic acid and lipid metabolic processes, while the expression of previously identified targets upon *Hnf1b* ablation, including cystic disease genes, was weakly or not affected. Postnatal analyses revealed renal abnormalities, ranging from glomerular cysts to hydronephrosis and, rarely, multicystic dysplasia. Urinary proteomics uncovered a particular profile predictive of progressive decline in kidney function and fibrosis, and displayed common features with a recently reported urine proteome in an RCAD pediatric cohort. Altogether, our results show that reduced HNF1B levels lead to developmental disease phenotypes associated with the deregulation of a subset of HNF1B targets. They further suggest that this model represents a unique clinical/pathological viable model of the RCAD disease.

## Abstract (precis)

**Summary:** A novel established mouse model carrying a heterozygous splicing human mutation in the *Hnf1b* gene exhibits phenotypes similar to those of patients with renal cysts and diabetes disease.

## INTRODUCTION

The transcription factor HNF1B is an important regulator of early mouse kidney, liver and pancreas organogenesis (Coffinier et al., 2002; De Vas et al., 2015; Gresh et al., 2004; Haumaitre et al., 2005; Heliot et al., 2013; Lokmane et al., 2008, 2010; Massa et al., 2013). Heterozygous mutations in the *HNF1B* gene are the cause of a complex human syndrome known as renal cysts and diabetes (RCAD; OMIM #137920), characterized by early onset of diabetes and developmental abnormalities of the kidney, genital tract and pancreas, as well as a variety of renal, liver, pancreas and biliary dysfunctions (Clissold et al., 2015; Barbacci et al., 2004; Bellanné-Chantelot et al., 2004, 2005; Haldorsen et al., 2008; Haumaitre et al., 2006; Kettunen et al., 2017; Lindner et al., 1999).

More than 150 *HNF1B* heterozygous mutations have been described, including missense, nonsense, insertion/deletions, frameshift and splice site mutations as well as whole-gene deletions (Alvelos et al., 2015; Barbacci et al., 2004; Bellanné-Chantelot et al., 2004; Chen et al., 2010; Edghill et al., 2006; Heidet et al., 2010). These mutations are either familial or *de novo* (40%). Intragenic mutations map predominantly in the DNA binding domain (exon-2 and exon-4), while whole-gene deletions (1.3 Mb deletion at chromosome 17q12) account for up to 50% of the *HNF1B* variants. *HNF1B* mutant carriers exhibit a highly variable phenotype, both between and within families. No clear genotype-phenotype correlations were observed for the type or location of mutations, and haploinsufficiency has been the main underlying disease proposed mechanism.

The most consistent clinical feature associated with heterozygous *HNF1B* mutations is severe non-diabetic renal disease. *HNF1B* mutations are also the most common monogenic causes of developmental kidney disease, being part of a spectrum of malformations known as congenital anomalies of the kidney and urinary tract (Nakayama et al., 2010) and autosomal dominant tubule interstitial kidney diseases (Devuyst et al., 2019). A large spectrum of renal abnormalities has been reported, including unilateral or bilateral cysts, multicystic dysplasia, oligomeganonephronia, hypoplastic glomerulocystic kidney disease, solitary kidney and various dysfunctions (Chen et al., 2010; Clissold et al., 2015; Ferrè and Igarashi, 2018; Shao et al., 2020).

The molecular mechanisms by which heterozygous mutations in *HNF1B* cause this broad spectrum of clinical symptoms remain poorly understood. Unlike humans, mice heterozygous for an *Hnf1b* null allele have no apparent phenotype, while the homozygous deletion results in early embryonic death (Barbacci et al., 1999). Constitutive inactivation of *Hnf1b* in the mouse epiblast (Haumaitre et al., 2005; Lokmane et al., 2008, 2010) or specific inactivation of *Hnf1b* either in renal tubules, liver or pancreas (Coffinier et al., 2002; De Vas et al., 2015; Desgrange et al., 2017; Gresh et al., 2004; Heliot et al., 2013) results in early embryonic or perinatal death, along with far more severe phenotypes than those observed in the context of the RCAD disease.

During renal development, *Hnf1b* has been shown to be required for early ureteric bud (UB) branching and the induction of nephrogenesis (Lokmane et al., 2010). Specific deletion either in nephron progenitors or in collecting ducts uncovered additional later functions in early nephron segmentation (Heliot et al., 2013; Massa et al., 2013) and normal patterning and epithelial organization of collecting ducts (Desgrange et al., 2017). Additionally, *Hnf1b* deletion in medullar tubules at relatively later stages resulted in cystic kidneys after birth and downregulation of several cystic disease genes, including the HNF1B direct targets *Pkhd1*, *Pkd2*, *Kif12* and *Umod* (Gresh et al., 2004; Hiesberger et al., 2005). These findings led to the hypothesis that the renal phenotype in *HNF1B* mutant carriers could be attributed to the global inhibition of these cystic genes. However, analyses of fetuses carrying two different *HNF1B* mutations (Haumaitre et al., 2006) as well as of adult mutant carriers (Faguer et al., 2012) show that the cystic renal phenotype was not associated with decreased expression of the cystic disease genes identified in mice, implying that a more complex HNF1B transcriptional network underlies the human disease.

The genetic discrepancies between mouse models and human disease further suggest that mouse mutant models generated so far do not correctly represent the human mutations or, alternatively, that mice are less sensitive to haploinsufficiency. To explore these possibilities and obtain a more comprehensive view of HNF1B function in organ development and disease in the context of the whole animal, we generated a novel RCAD mouse model by introducing a previously identified human hotspot mutation at the intron-2 splice donor site (<IVS2nt+1G>T) (Bingham et al., 2003; Harries et al., 2004). Moreover, because patients with these mutations exhibited the typical features of *HNF1B* mutations (Bingham et al., 2003), this model could be representative of a large number of *HNF1B* intragenic disease-causing mutations.

Characterization of the renal phenotype showed that mouse heterozygous mutants [referred to as splicing mutation intron-2 (Sp2) *Hnf1b^Sp2/+^*] exhibited bilateral cysts and tubular dilatations as well as glomerular cysts from embryonic day (E)15, along with rare cases of genital tract abnormalities and extrarenal manifestations similar to those described in human *HNF1B* mutant carriers. Unlike previous mouse mutants heterozygous for a null allele, in which the HNF1B protein levels remained either unchanged or even increased (Kornfeld et al., 2013), the HNF1B protein levels of *Hnf1b^Sp2/+^* heterozygous mutants were reduced by 30-40%.

Transcriptional profiling analyses indicated that only a subset of the HNF1B target genes expressed primarily in early and mature proximal tubules (PTs) was sensitive to reduced HNF1B levels at different embryonic stages, whereas the previously identified targets upon *Hnf1b* ablation were relatively insensitive. Postnatal analyses revealed several renal abnormalities, ranging from few clusters of glomerular cysts and microcysts to hydronephrosis and, rarely, multicystic dysplasia. Further urine proteomic analyses uncovered a particular signature of differentially excreted peptides in *Hnf1b*^Sp2/+^ mice, exhibiting several similarities to the urinary peptide signature reported in pediatric RCAD patients (Ricci et al., 2019).

Our results highlight that reduced *Hnf1b* dosage in this mouse model differentially affects the expression of target genes, leading to the onset of disease phenotypes.

## RESULTS

### Generation of a mouse model reproducing a human *HNF1B* splicing mutation

A mouse model carrying a point mutation at the intron-2 splice donor site was generated by homologous recombination by introducing a G to T point mutation (Fig. S1A), thus reproducing the c.544+1G>T (<IVS2nt+1G>T) human mutation (Bingham et al., 2003). The resulting *Hnf1b* mutated allele encompassed the human splicing point mutation and a unique LoxP site within intron-1.

To define the consequences of this mutation on *Hnf1b* mRNA processing, we initially performed semiquantitative real-time PCR (RT-PCR) using RNA from *Hnf1b*^Sp2/+^ heterozygotes and wild-type (WT) kidneys and primers located in exon-1 and exon-3. Sequence of the PCR products in *Hnf1b*^Sp2/+^ mutants indicated the production of the two expected *Hnf1b* spliced isoforms A and B and four additional novel transcripts, present at low levels, corresponding to the isoforms A and B in which were deleted either exon-2 or the last 32 bp of exon-2 through the activation of a near cryptic splice donor site (Fig. S1B; see Materials and Methods). The same pattern of alternative splicing in *Hnf1b*^Sp2/+^ heterozygous mice was observed at different stages (Fig. S1D).

Notably, previous mRNA analysis has shown that the human splicing mutations IVS2nt+2insT and IVS2nt+1G>T also resulted in exon-2 skipping. However, despite the conservation of the exon-2 cryptic splice donor site in humans (Fig. S1C), variants lacking the last 32 bp of exon-2 have not been described (Harries et al., 2004).

The predicted consequences of deleting either the entire or part of exon-2 are the generation of a frameshift leading to premature stop codons deleting the DNA binding domain and the entire C-terminal transactivation domain. This indicated that the spliced mutant transcripts are expected to generate non-functional proteins.

We subsequently examined the levels of *Hnf1b* transcript and protein produced by the normal allele in *Hnf1b^Sp2/+^* heterozygous mutants and WT littermates, and searched for the presence of putative truncated proteins produced by the mutant allele.

**Fig. 1.**
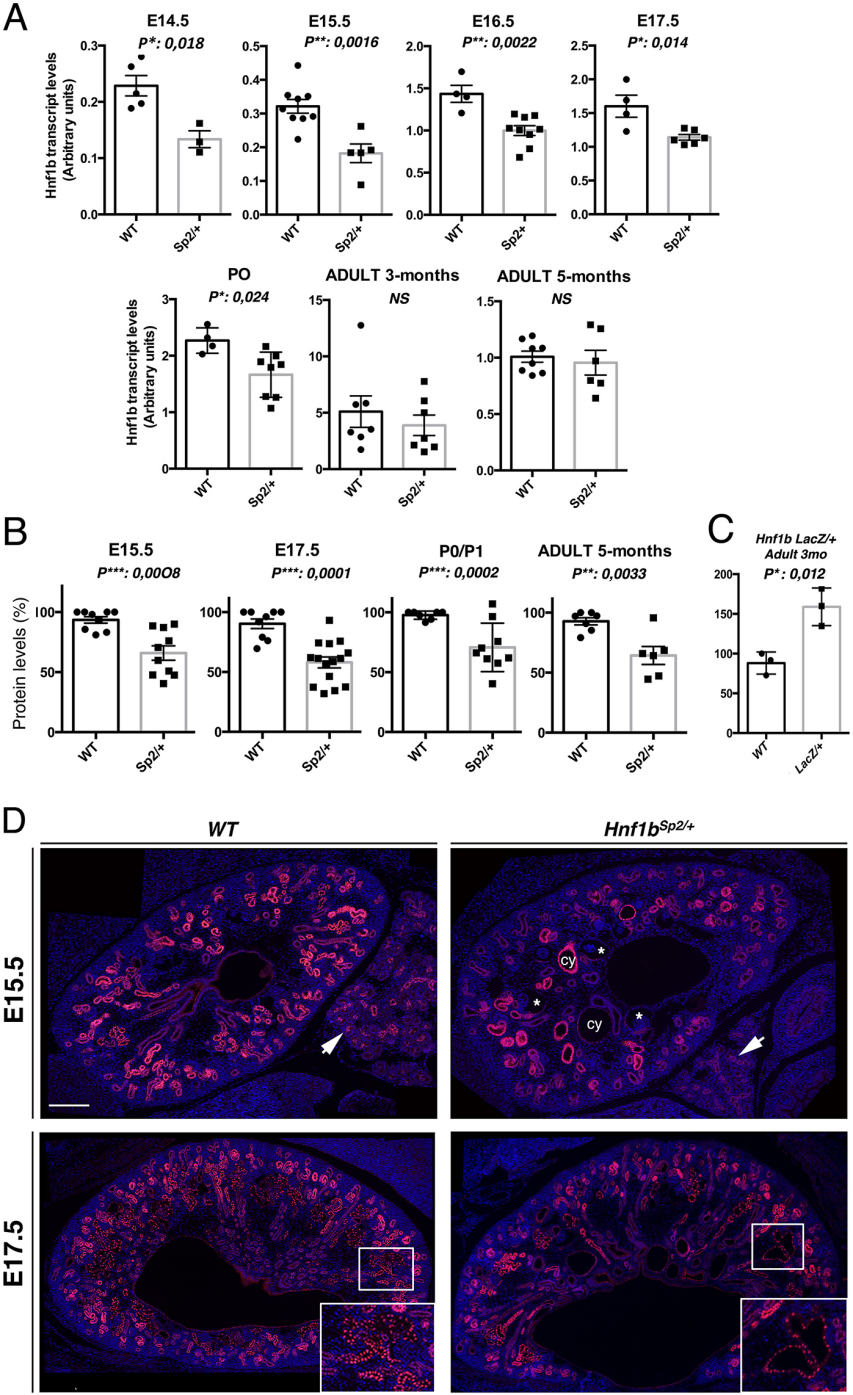
**Expression levels of normal *Hnf1b* transcripts and protein from WT and *Hnf1^Sp2/+^* heterozygous mutants.** (A) qRT-PCR of normal *Hnf1b* transcripts in WT and *Hnf1b^Sp2/+^* kidneys at the indicated stages. WT versus *Hnf1b^Sp2/+^* sample numbers were as follows: E14.5, *n*=5 vs *n*=3*;* E15.5, *n*=9 vs *n*=5; E16.5, *n*=4 vs *n*=9; E17.5, *n*=4 vs *n*=6; P0, *n*=4 vs *n*=8; adult 3 months, *n*=7 vs *n*=7; adult 5 months, *n*=8 vs *n*=6. Significant decreases in *Hnf1b* transcript relative to WT were at E14.5 (58.5%), E15.5 (57%), E16.5 (50%) and P0 (64%). NS, not significant. (B) Western blot quantification of HNF1B protein levels in *Hnf1b^Sp2/+^* relative to WT*.* Significant decreases to 70%, 62%, 72% and 69% relative to WT were observed at E15.5, E17.5, P0 and 5 months, respectively. (C) Western blot quantification of 3-month-old WT and heterozygous *Hnf1b^LacZ^*^/+^ show a 98% increase in HNF1B levels in the mutants. Error bars represent s.e.m. Unpaired Student's *t*-test, *P<0.05, **P<0.01, ***P<0.001. (D) HNF1B immunostaining of E15.5 and E17.5 embryo sections show, in *Hnf1b^Sp2/+^* kidneys, a global decrease in the number of HNF1B^+^ structures, together with decreased nuclear staining in some regions (inset in E17.5). Note adjacent pancreatic ducts exhibiting decreased HNF1B expression (arrows), glomerular cysts (asterisks), tubular dilatations (cy) and HNF1B nuclear staining in both non-dilated and dilated renal tubules. Scale bar: 200 μm.

*Hnf1b* transcript levels were examined by quantitative RT-PCR (qRT-PCR) using primers located in the ATG translational site and the last 32 bp of exon-2, thus allowing the detection of transcripts produced only by the normal *Hnf1b* allele (Table S2). We found that, in *Hnf1b^Sp2/+^*, the *Hnf1b* transcripts were significantly decreased by 30-42% relative to WT levels, in particular during embryonic stages. In adults, the reduction in transcript levels was highly variable and modest without reaching significance (Fig. 1A).

To examine the HNF1B proteins produced by both the normal and mutant allele in *Hnf1b^Sp2/+^* heterozygous mice, we performed western blot analyses using whole-cell extracts from microdissected kidneys of several independent embryo litters or adult mice and anti-HNF1B antibodies raised against residues located within the first exon. As controls, we used extracts from transfected cells with expression vectors of either full-length HNF1B variant A or the putative truncated proteins encoded by spliced mutant transcripts.

These analyses showed the lack of any detectable truncated protein encoded by abnormally *Hnf1b* spliced transcripts at any stage examined and even upon increasing 4-fold the concentration of extracts, under conditions in which the truncated spliced versions expressed in transfected cells were easily detected (Fig. S2A-C,E). Thus, these truncated proteins are either very unstable or not produced as previously described in several dominant mutations associated with premature termination codons (PTCs) (see Discussion). Unlike previously characterized heterozygous mutants, we found a significant decrease in HNF1B protein levels in the *Hnf1b^Sp2/+^* mutants, not only at embryonic stages but also in adults (Fig. 1B), further suggesting a post-transcriptional and/or translational control of HNF1B in postnatal life. By contrast and consistent with the absence of phenotype, we confirmed that, in *Hnf1b^Lacz/+^* heterozygous mice, HNF1B protein levels were significantly increased (Kornfeld et al., 2013) (Fig. 1C)*.*

To acquire a detailed spatial pattern of HNF1B expression in heterozygous mutants, we performed immunostainings at different embryonic stages. Compared to WT, *Hnf1b^Sp2/+^* mutants exhibited from E15.5 a global reduction of the structures positive for HNF1B, together with an unequal decrease in nuclear staining in several regions (highlighted in Fig. 1D insets). Dilated tubules did still exhibit nuclear staining (Fig. 1D).

In conclusion, these results show that the splicing mutation results in a loss-of-function allele together with decreased levels of the functional HNF1B protein compared with WT levels. Notably, HNF1B protein is not decreased homogeneously in expressing structures, suggesting that these variations occur stochastically and underlie the heterogeneity in the disease phenotype.

### *Hnf1b^Sp2/+^* heterozygous mutant embryos exhibit bilateral glomerular cysts, tubular dilatations and hydronephrosis with variable severity depending on the genetic background

**Fig. 2.**
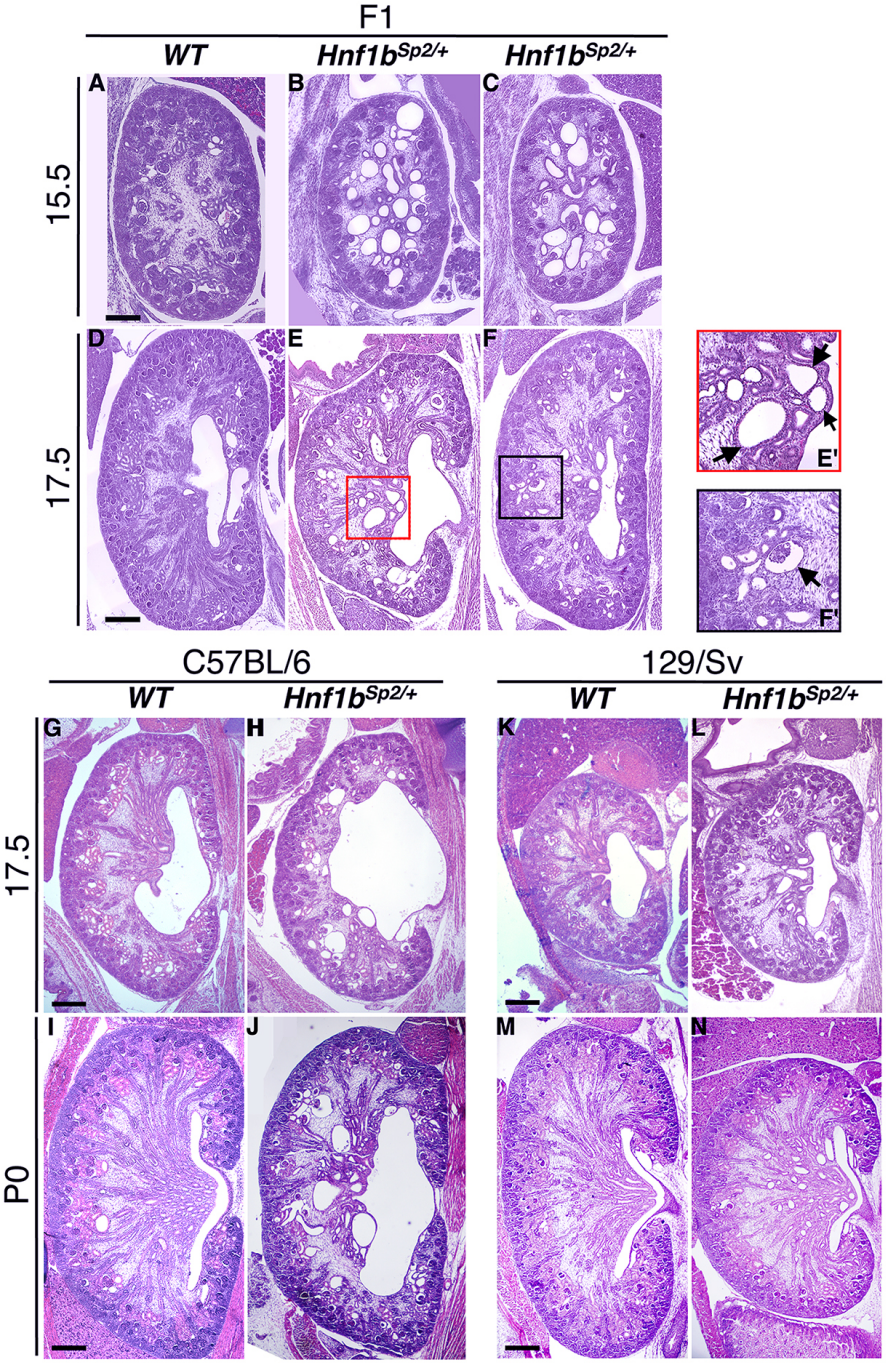
**Histology of *Hnf1b^Sp2/+^* embryos from different genetic backgrounds highlights the early onset of renal cysts and hydronephrosis.** (A-F) Representative Hematoxylin and Eosin (H&E)-stained sections of embryo kidneys show glomerular cysts and tubular dilatations in the medulla at E15.5 and E17.5 (arrows in E′, higher magnification of E). Arrow in F′ (higher magnification of F) shows cystic glomeruli. Embryos were from a mixed background (F1). (G-N) Inbreeding onto C57BL/6N and 129/sv backgrounds showed different phenotype severities. Note in the C57BL/6N background, pelvic dilatations at E17.5 (H), hydronephrosis and duplicated kidney at P0 (H,J) (see also Fig. S4), in addition to cystic glomeruli and medullar tubules dilatations (H,J), also observed in the 129/sv background (L,N). Images are representative of *n=*6 for each genotype. Scale bars: 200 μm.

To assess the onset and progression of the renal phenotype, we performed histological analyses of heterozygous mutants and WT littermates at different embryonic stages initially in a C57BL/6N×129/sv mixed background (F1). Up to E14.5, heterozygous embryos developed apparently normally (Fig. S3A), although in some cases mutant embryo kidneys exhibited dilated Bowman's capsules (Fig. S3C). Subsequently, from E15.5, we reproducibly observed bilateral cysts, including glomerular and tubular cysts in the cortico-medullar regions (Fig. 2 compare A with B,C). As kidney growth progressed, from E17.5, there was an apparent reduction in the volume of cystic structures relative to whole kidney volume, but glomerular cysts exhibited a rather similar distribution throughout development (Fig. 2D-F).

We then examined the influence of mouse strain susceptibility on the disease phenotype by backcrossing the *Hnf1b^Sp2/+^* mutant mice into two inbred backgrounds: C57BL/6N and 129/sv. As observed in the mixed background, mutant kidneys in these two backgrounds exhibited tubular dilatations and glomerular cysts from E15.5 (Fig. 2G-N) and a more severe renal phenotype at embryonic stages than in postnatal life. However, in the C57BL/6N background, the *Hnf1b^Sp2/+^* embryonic kidneys presented a relatively more severe and variable phenotype, particularly at later stages and in newborn pups, with higher numbers of both medullar and glomerular cysts, frequent unilateral or bilateral pelvic dilatations and hydronephrosis and, more rarely, duplicated kidneys (Fig. 2; Fig. S4). A summary of histological renal phenotypes observed from E18.5 to postnatal day (P)0 is shown in Table S1A.

Heterozygous mutant embryos or newborns were obtained at the expected Mendelian ratio in different backgrounds. However, in the C57BL/6N background, 10-15% of heterozygous mutants died between P1 and P25 (Table S1B). Heterozygous mutants in both backgrounds were able to reproduce, but they were less fertile than WT littermates. Further postnatal analysis revealed rare cases of genital tract abnormalities (agenesis of the uterine horn, epididymis cysts, abnormal branched and highly dilated seminal vesicles) in either C57BL/6N or 129/sv backgrounds. Heterozygous mutants also exhibited several pancreatic dysfunctions, including glucose intolerance and pancreatitis, a phenotype that will be described elsewhere (Quilichini et al., 2021). Unless otherwise indicated, subsequent analyses were performed either in a mixed or in the C57BL/6N background.

These data show that *Hnf1b^Sp2/+^* heterozygous mutants exhibited several of the urogenital phenotypes described in *HNF1B* mutant carriers. They also suggest that genetic modifiers may either aggravate (C57BL/6N) or attenuate (129/sv) the phenotype, further contributing to variability in phenotypic manifestations.

### *Hnf1b^Sp2/+^* embryos display early PT dilatations together with delayed differentiation of PTs

**Fig. 3.**
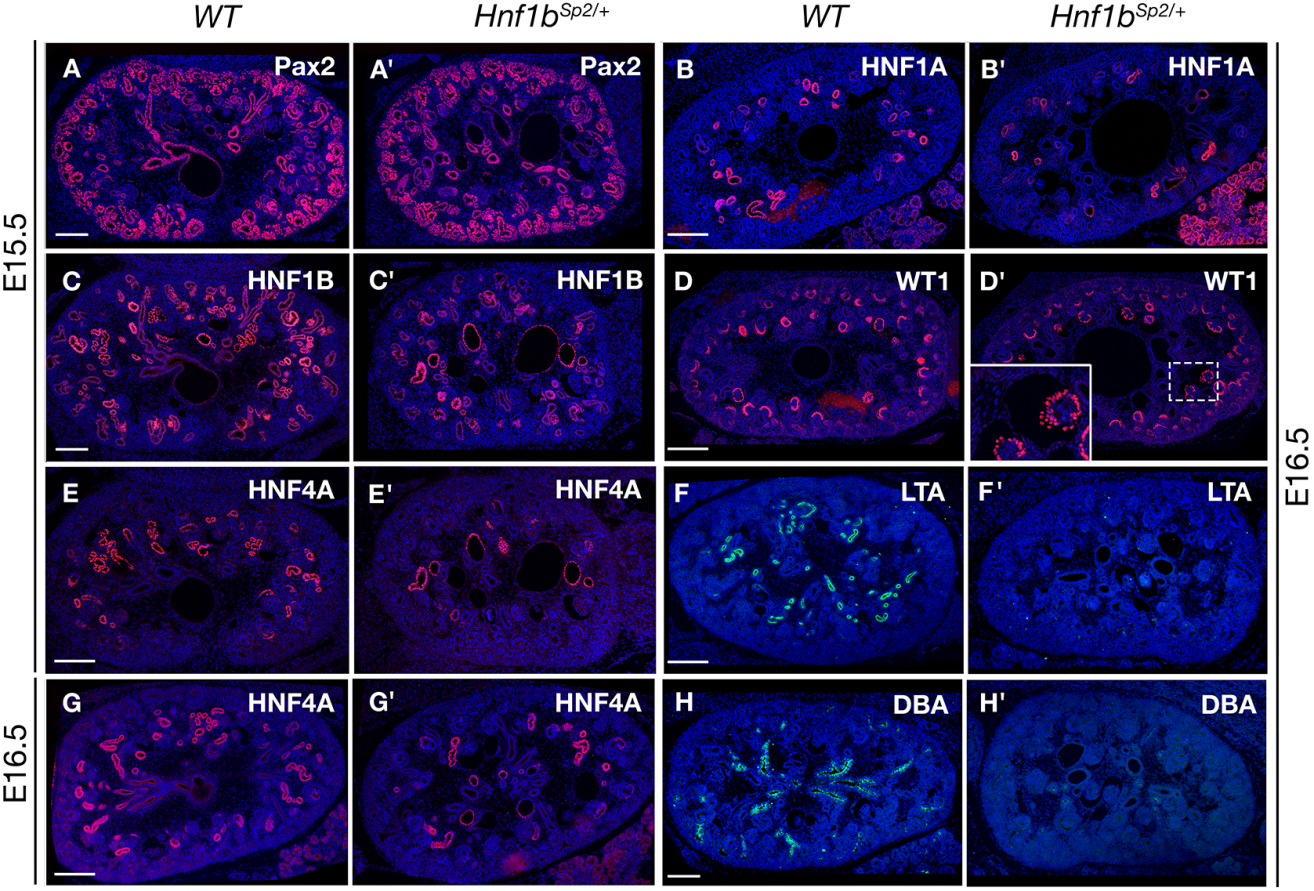
***Hnf1b^Sp2/+^* embryo kidneys exhibit normal ureteric bud branching but glomerular cysts and delayed proximal tubule (PT) differentiation.** (A-H′) Immunohistochemical analysis of WT and *Hnf1b^Sp2^*^/+^ embryo kidneys with Pax2 (A,A′), HNF1B (C,C′), the PT markers HNF4A (E,E′,G,G′), HNF1A (B,B′) and LTA (F,F′), WT1 (D,D′, inset shows glomerular cyst magnification with partially disorganized podocyte expression, and the collecting duct lectin DBA (H,H′) at the indicated stages. Note in *Hnf1b^Sp2/+^* sections decreased HNF4A^+^ PT structures (E′,G′) and PT dilatations, particularly at E15.5 (E′), which correlate with HNF1B expression in a serial section (C′) of the same embryo, decreased HNF1A PT expression together with increased acinar pancreatic expression (B′), and the absence of lectins LTA (F′) and DBA (H′). Sections are co-stained with 4′,6-diamidino-2-phenylindole (DAPI). Scale bars: 200 μm.

**Fig. 4.**
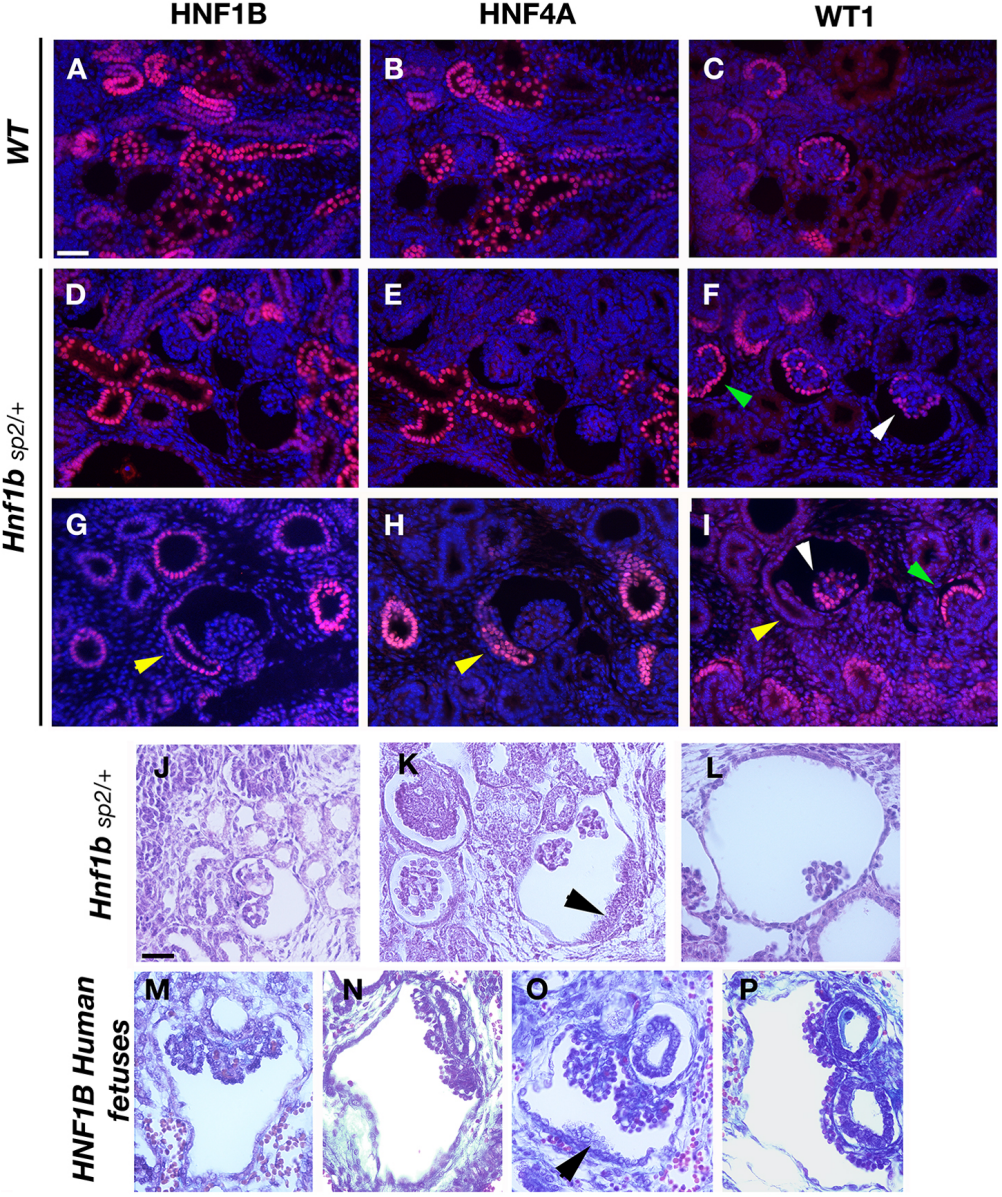
**Glomerular cyst development, progressive tuft atrophy and abnormal PT implantation in *Hnf1b^Sp2^*^/+^ embryos.** (A-I) Histological comparison with human *HNF1B* mutant fetuses. Immunohistochemical analysis of E16.5 WT (A-C) and *Hnf1b^Sp2^*^/+^ embryos (D-I). PTs are highlighted by the co-expression of HNF4A and HNF1B; developing glomeruli and condensed mesenchyme are shown by WT1 staining. Sections are co-stained with DAPI. Note that HNF4A staining in *Hnf1b^Sp2^*^/+^ (E,H) was intentionally increased to better visualize positive PTs. Note in *Hnf1b^Sp2^*^/+^ dilated PTs that were HNF4A^+^ (E,H), glomerular cysts with disorganized podocyte layer stained by WT1 (white arrowheads); non-cystic glomeruli show normal podocyte layer (green arrowheads). Yellow arrowheads (H,I) show abnormal lateral insertion of the glomerulotubular junction into Bowman's capsule and disorganized HNF4A expression (H). (J-P) H&E-staining of E17.5 *Hnf1b^Sp2^*^/+^ embryos (J-L) and human mutant fetuses described in Haumaitre et al. (2006) (M-P). Black arrowheads indicate disorganized layer cells in mutants and human fetuses (K,O). Note also collapsed or disrupted tufts inside highly widened Bowman's capsules (L,P). Scale bars: 50 μm.

*Hnf1b* is known to be required for early UB branching and initiation of nephrogenesis (Lokmane et al., 2010). However, in *Hnf1b^Sp2/+^* mutants, neither early UB branching nor the expression of the early targets identified upon *Hnf1b* ablation were affected (Fig. S3B). Notably, the expression of Pax2, the transcription of which in the collecting system depends on *Hnf1b* (Desgrange et al., 2017; Lokmane et al., 2010; Paces-Fessy et al., 2012), was not affected in *Hnf1b^Sp2/+^* embryos at E14.5 or E15.5 (Fig. 3A,A′; Fig. S3B), or at later stages (Fig. S3C). Nascent nephrons appeared normally induced as indicated by the presence of normally shaped comma- and S-shaped bodies (Fig. S3B) and WT1-stained glomerular podocytes (Fig. 3D,D′). Although normally expressed both in the condensed mesenchyme around the UBs and podocytes, WT1 expression was disrupted in glomeruli with dilated Bowman’s capsules or cystic glomeruli (Fig. 3D′; see below, Fig. 4F,I).

We then examined the origin of cystic/dilated tubular structures by staining with different nephron and collecting duct markers from E15.5/E16.5 (Fig. 3) to E17.5 and P0 (Fig. S5). Mutant kidneys without overt hydronephrosis were examined to visualize the collecting duct network and medullar nephron tubules. The expression of HNF4A, a marker of early PTs and a target of HNF1B (Heliot et al., 2013), was heterogeneously and severely decreased at E15.5 and E16.5 (Fig. 3E′,G′; see also Figs S5 and S8), suggesting defective differentiation of PTs. Notably, HNF4A staining, which correlated with the reduced levels of HNF1B (compare Fig. 3C′,D′), uncovered that a fraction of PTs were highly dilated, particularly at E15.5.

The expression of HNF1A, restricted to mature PTs, was similarly reduced at E16.5 (Fig. 3B′). Its expression was completely restored by E17.5 (Fig. S3C), in contrast to HNF4A expression, which showed only a partial restoration at E17.5 (Fig. S5A′) or P0 (Fig. S5F′). Consistent with these observations, the mature PT marker LTA was not expressed up to E16.5 (Fig. 3F′) and began to be expressed by E17.5 (Fig. S5B′). However, *Hnf1b^Sp2/+^* PT clusters that were LTA^+^ both at E17.5 and P0 exhibited an unequal distribution and remained reduced in size (Fig. S5B′,G′). Interestingly, the Na-K-Cl co-transporter (NKCC2; SLC12A1), expressed in the thick ascending limb (TAL) of the loop of Henle, stained most of *Hnf1b^Sp2/+^* medullar cysts and tubular dilatations at E17.5 and P0 (Fig. S5C′,H′). Distal tubules labeled by SLC12A3 (NCC) showed only rare and mild dilatations (data not shown).

*Hnf1b^Sp2/+^*collecting ducts, stained either by AQP2 (Fig. S5D′,I′) or pancytokeratin (CK) (Fig. S5E′), were mainly devoid of dilatations. Intriguingly, they were not stained by Dolichos biflorus lectin (DBA), either during development or in postnatal life (Fig. 3H,H′; Fig. S5J′), uncovering impaired polarization of glycoconjugates in mutant collecting duct cells. This observation evoked an altered differentiation status of collecting duct cells, because previous studies have shown that UB cells begin to express DBA-binding glycoconjugates once they differentiate into stalks (Michael et al., 2007).

Thus, in addition to glomerular cysts, *Hnf1b^Sp2/+^* heterozygous mutants display tubular cysts and dilatations that predominate initially in early PTs and subsequently from E17.5 in the TAL of the loop of Henle. Collecting ducts appeared morphologically normal but failed to express DBA-binding glycoconjugates.

### Glomerular cysts display abnormal glomerulotubular insertion in *Hnf1b^Sp2/+^* developing kidneys

Reminiscent of the glomerulocystic kidney disease described in *HNF1B* human mutant carriers (Bingham et al., 2001), glomerular cysts observed from E15 throughout development are the most common and earliest feature of the renal phenotype of *Hnf1b*^Sp2/+^. It has been proposed that glomerular cystogenesis could secondarily result from transient obstruction of the urinary tract or developing nephrons or from defects in the junction of the PTs to the glomeruli (glomerulotubule junction) (Lennerz et al., 2010).

Without evidence of urinary tract obstruction, we further examined HNF1B, HNF4A and WT1 immunostainings on serial embryonic sections, focusing on glomeruli with different degrees of Bowman's capsule expansion. We reproducibly observed that cystic glomeruli were surrounded by a decreased number of PTs that were often dilated or cystic (Fig. 4 compare A,B with D,E,G,H). Additionally, HNF4A and HNF1B staining showed that the glomerulotubular junction was laterally inserted into the Bowman's capsule and exhibited an unequal disorganized expression of HNF4A (Fig. 4H). Immunodetection of WT1 showed that the layer of podocytes of developing glomeruli with expanded Bowman's capsules became progressively disorganized, whereas *Hnf1b^Sp2/+^* non-cystic glomeruli displayed the normal layer of podocytes (Fig. 4F,I). In more advanced glomerular cysts, the expression of WT1 was lost (Fig. S11).

Analysis of two *HNF1B* human mutant fetuses showing a very severe cystic renal phenotype (Haumaitre et al., 2006) also exhibited glomerulotubule junction defects [Fig. 4M,N (R112fs mutant fetus, 27 weeks), O,P (P472fs mutant fetus, 31.5 weeks)]. They also presented separated tufts, into two to five, whereas in *Hnf1b^Sp2/ +^* mice, tufts were separated into two or three or not separated (Fig. 4J-L).

These data together suggest that glomerular cysts in *Hnf1b^Sp2/+^* mice very likely result from early defects in PT differentiation, affecting glomerulotubule insertion and leading to the accumulation of glomerular filtrate, with subsequent expansion of the Bowman's space.

### *Hnf1b^Sp2/+^* cysts and tubular dilatations of developing nephrons are associated with defects in nephron differentiation and apico-basal polarity

**Fig. 5.**
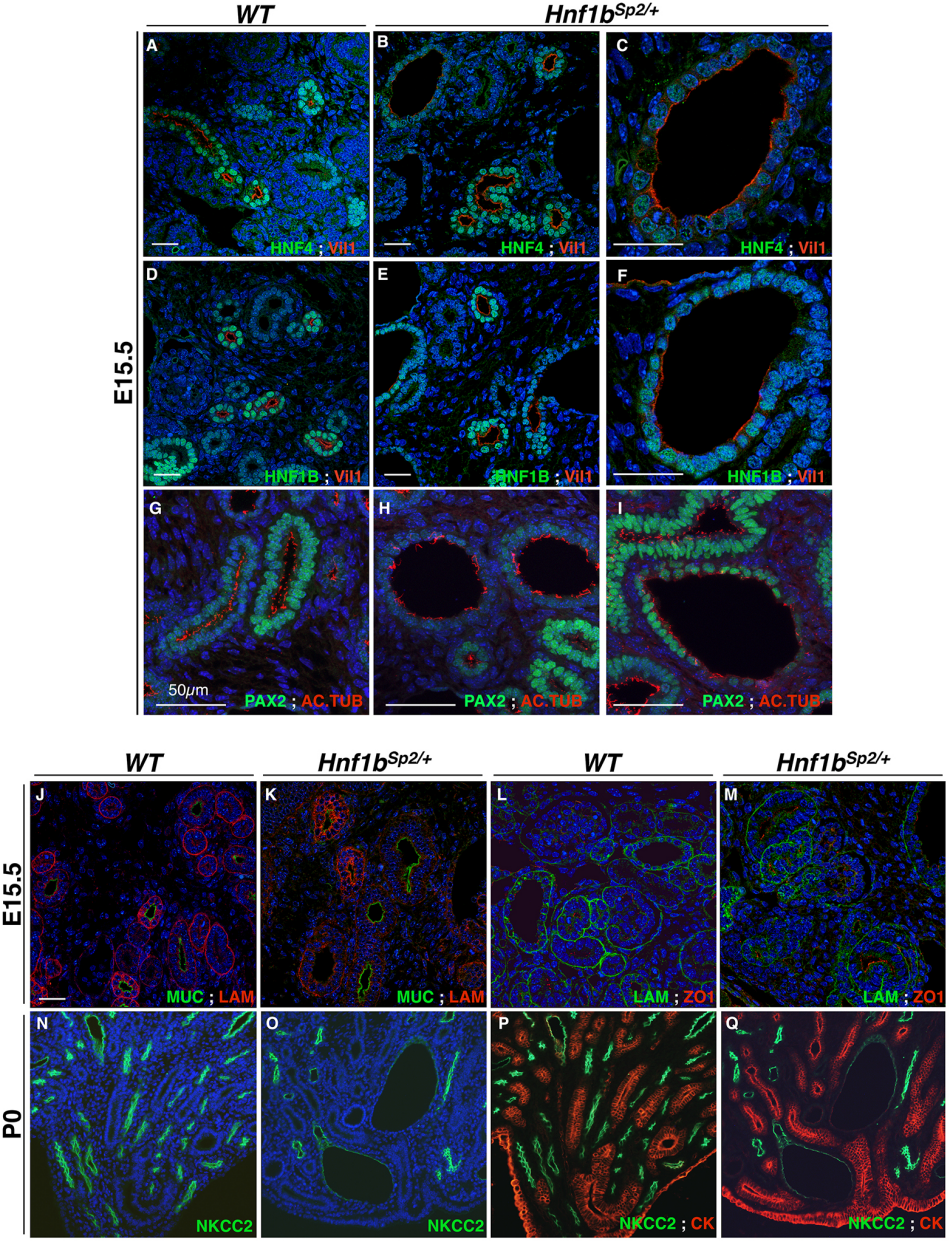
**Cystic and dilated PT and thick loop of Henle cells exhibit abnormal apico-basal polarity and reduced number of cilia.** (A-I) Confocal microscopy of co-stained sections with Vil1-HNF4A and Vil1-HNF1B, revealed that Vil1 expression (A-F), localized at the PT brush border, is interrupted in several regions of *Hnf1b^Sp2/+^* cystic structures, indicative of brush-border loss (magnifications in C and F). Confocal images of acetylated tubulin staining show some regions with cells devoid of primary cilia in E15.5 *Hnf1b^Sp2/+^* cystic PTs, while non-dilated tubules have apparent normal cilia distribution compared with WT (compare H,I with G). (J-M) Confocal images of Muc1-Lam1 and Lam1-ZO1 co-immunohistochemistry show that the collecting duct apical marker Muc1 in E15.5 *Hnf1b^Sp2/+^* (K) is expressed similar to WT (J), while the basement membrane Lam1 exhibits a partially disorganized pattern (K,M) compared to WT kidneys (J,L). The tight junction protein ZO1 (L,M) is normally expressed. (N-Q) Co-stained P0 kidney sections with NKCC2 (N,O) and merged NKCC2-CK (P,Q) show decreased staining of NKCC2 in *Hnf1b^Sp2/+^*cystic medullar loops of Henle (O,Q) compared with WT (N,P). Apical CK expression in collecting ducts (P,Q) is not affected. Confocal images were captured with a ×20 objective (A-M). Optical pictures were captured with a ×20 (N-Q) objective. Scale bars: 50 μm.

Cystogenesis in embryonic kidneys has been reported to be associated with several cellular defects, including abnormal cell polarity, primary cilia defects, changes in cell–cell and cell–matrix interactions, as well as increased proliferation and apoptosis (Wilson, 2011). We found that the PT brush border marker Vil1 (Villin) was normally expressed in *Hnf1b^Sp2/+^* non-dilated tubules, but it was interrupted in cystic PTs (Fig. 5 compare A,D with B,C,E,F). Cystic PTs also exhibited a stronger decrease in HNF4A expression (Fig. 5 compare A with B,C). The expression of HNF1B was also moderately decreased in these dilated PTs compared to its expression in other renal tubules (Fig. 5E,F).

Staining with α-acetylated tubulin to visualize cilia showed that the number of cells with cilium lining non-dilated *Hnf1b^Sp2/+^* collecting ducts or nephron tubules was not significantly different from that of WT embryos. However, we observed fewer cells with cilia in cystic and dilated PTs in comparison to non-dilated PTs (Fig. 5 compare G and H,I).

Interestingly, the basal membrane marker laminin 1 (Lam1) exhibited a global decrease and partial disorganization in E15.5 heterozygous kidneys, both in dilated and non-dilated tubules (Fig. 5K,M). In medullar cystic tubules, the NKCC2 apical staining of epithelial cells of the TAL of the loop of Henle was also decreased in a proximo-distal gradient and disorganized (Fig. 5O,Q), whereas the apical markers of collecting ducts, Muc1 and CK, were both correctly expressed (Fig. 5J,K,P,Q).

Further analysis of proliferation, using the mitosis marker phosphorylated histone H3, showed comparable numbers of proliferating cells in E14.5 renal tubules of heterozygous mutants and WT (Fig. S6A). However, at later stages, the number of proliferating cells in *Hnf1b^Sp2/+^* normal tubular structures exhibited a 1.84-fold increase relative to WT tubules. Consistent with the enhanced proliferation associated with cystic expansion in autosomal dominant polycystic kidney disease (Hopp et al., 2012), a higher and significant increase in proliferating cells was observed in *Hnf1b^Sp2/+^* dilated/cystic tubules relative to *Hnf1b*^Sp2/+^ non-dilated normal tubules (Fig. S6B,C). No changes were observed in the number of apoptotic cells assessed by terminal deoxynucleotidyl transferase dUTP nick-end labeling (TUNEL) (not shown).

Together, these results show that decreased levels of HNF1B appear to affect basal membrane organization without affecting apical cell polarity markers in non-dilated tubules. Moreover, proliferation was increased in cystic tubules relative to non-dilated tubules, and even non-dilated tubules exhibited increased proliferation compared with WT. The decreased expression of apical and brush border markers, as well as the decreased number of cilia observed in *Hnf1b^Sp2/+^* cystic tubules, appear to be secondary to tubular dilatations.

### Transcriptomic analyses of heterozygous mutant kidneys during development uncover variable dosage sensitivity among *Hnf1b* targets

To further elucidate the cellular and molecular components sensitive to HNF1B levels, we performed RNA sequencing (RNA-seq) on WT and heterozygous mutant kidneys at E14.5 (considered histologically as pre-disease kidneys) and at disease stages (E15.5, E17.5 and P1) as detailed in the Materials and Methods.

**Fig. 6.**
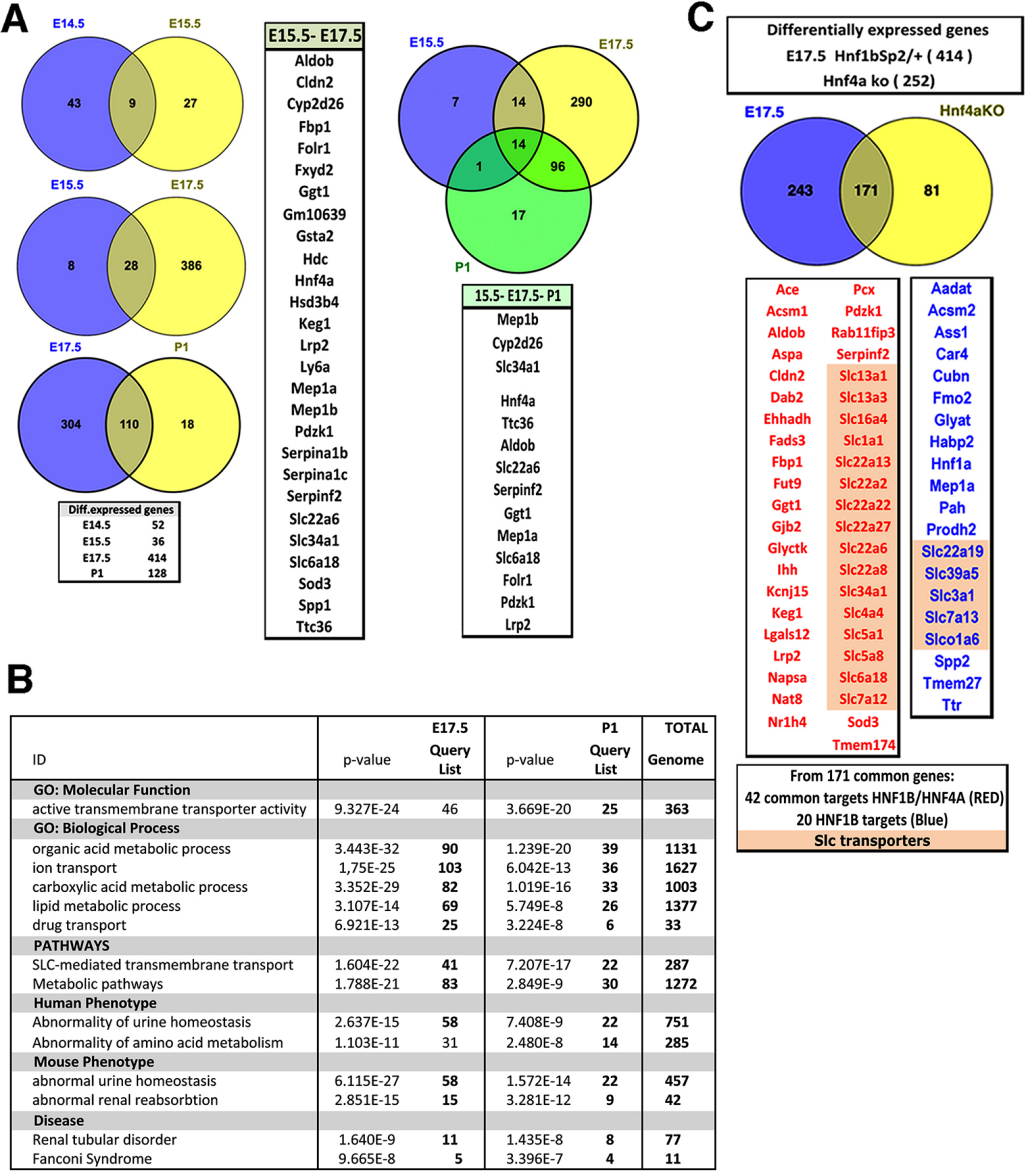
**Differential expression analysis of *Hnf1b^Sp2/+^* mutant versus WT kidneys at different embryonic stages.** (A) Venn diagram showing the overlap of genes differentially expressed from E14.5 to P0. (B) Gene ontology (GO) analysis of downregulated genes showing the top enriched terms (Table S4). (C) Venn diagrams show the overlap of differentially expressed genes of E17.5 *Hnf1b^Sp2/+^* and P0 *Hnf4a* knockout (KO) kidneys (Marable et al., 2020). Also indicated are HNF1 and HNF4 common target genes (red) and HNF1 targets (blue).

Because heterozygous mutants still expressed HNF1B, we considered an absolute fold-change cutoff value of > or <0.5 log2FC, with an adjusted *P*-value <0.05. Remarkably, several genes were differentially expressed with log2FC>−1 at all stages (Table S3), while only a few differentially expressed upregulated genes were detected. We found an increased number of downregulated genes from E14.5 to E17.5 followed by a partial decrease at P1, in agreement with a partial restoration of the expression of several PT markers. Accordingly, the numbers of downregulated genes shared between stages increased to reach 107 genes between E17.5 and P1 (Fig. 6A; Table S3, File 5).

Downregulated genes with greater fold changes were predominantly expressed in early and maturing PTs and to a lesser extent in primitive loops of Henle and distal tubules (Table S3, File 6). At E17.5 and P1, downregulated genes included drug-metabolizing enzymes (*Abcc2*, *Ggt1*, *Fmo2*, *Akr1c1*) and more than 50 SLC-transmembrane-transporter genes (organic cation, sodium glucose, sodium phosphate transporters; Table S3, Files 7 and 8).

Gene ontology (GO)-term analyses (Chen et al., 2009) highlighted transporter activity, active transmembrane transporter activity, organic acid and lipid metabolic processes and metabolism terms, as well as an association with abnormal renal/urinary system physiology, renal reabsorption, aminoaciduria and decreased urine osmolality. From E17.5, and consistent with the onset of mature cell types in the developing nephrons, the pathways enriched included SLC-active transmembrane transport (transport of glucose and small molecules and metabolism) (Fig. 6B,C; Table S4).

The regulatory sequences of most downregulated genes contained HNF1 consensus binding sites. Consistently, specific HNF1B recruitment to the regulatory sequences of many of them has been reported by chromatin immunoprecipitation (ChIP)-PCR or ChIP sequencing [reviewed by Ferrè and Igarashi (2018) and Shao et al. (2020)]. Amongst those targets strongly downregulated at most stages were *Hnf4a*, *Tmem27* (*Cltrn*), *Cubn*, *Spp1*, *Spp2*, *Pah*, *Kcnj1*, *Pdzk1* and several SLC-transmembrane transporter genes, representing the subset of targets that appeared particularly sensitive to reduced HNF1B protein levels (Table S3). Interestingly, many of the genes downregulated at E17.5/P1 and regulated by HNF1B were also found strongly decreased in *Hnf4a* mutant P1 kidneys (Marable et al., 2020) (Table S3, File 8). By contrast, the expression of previously identified targets strongly reduced upon *Hnf1b* ablation, including *Wnt9b*, *Pax2*, *Pkd2*, *Bicc* (*Bicc1*), *Tg737* (*Ift88*), *Crb3*, *Kif12*, *Cys1*, *Glis2* and *Glis3*, were either modestly decreased or not affected. These results were further validated by qRT-PCR (Fig. S7, Table S3). Interestingly, the well-known targets *Umod*, *Tmem27* and *Pkhd1* (Gresh et al., 2004) were found to be significantly downregulated at E17.5, but not at P1 (Table S3).

**Fig. 7.**
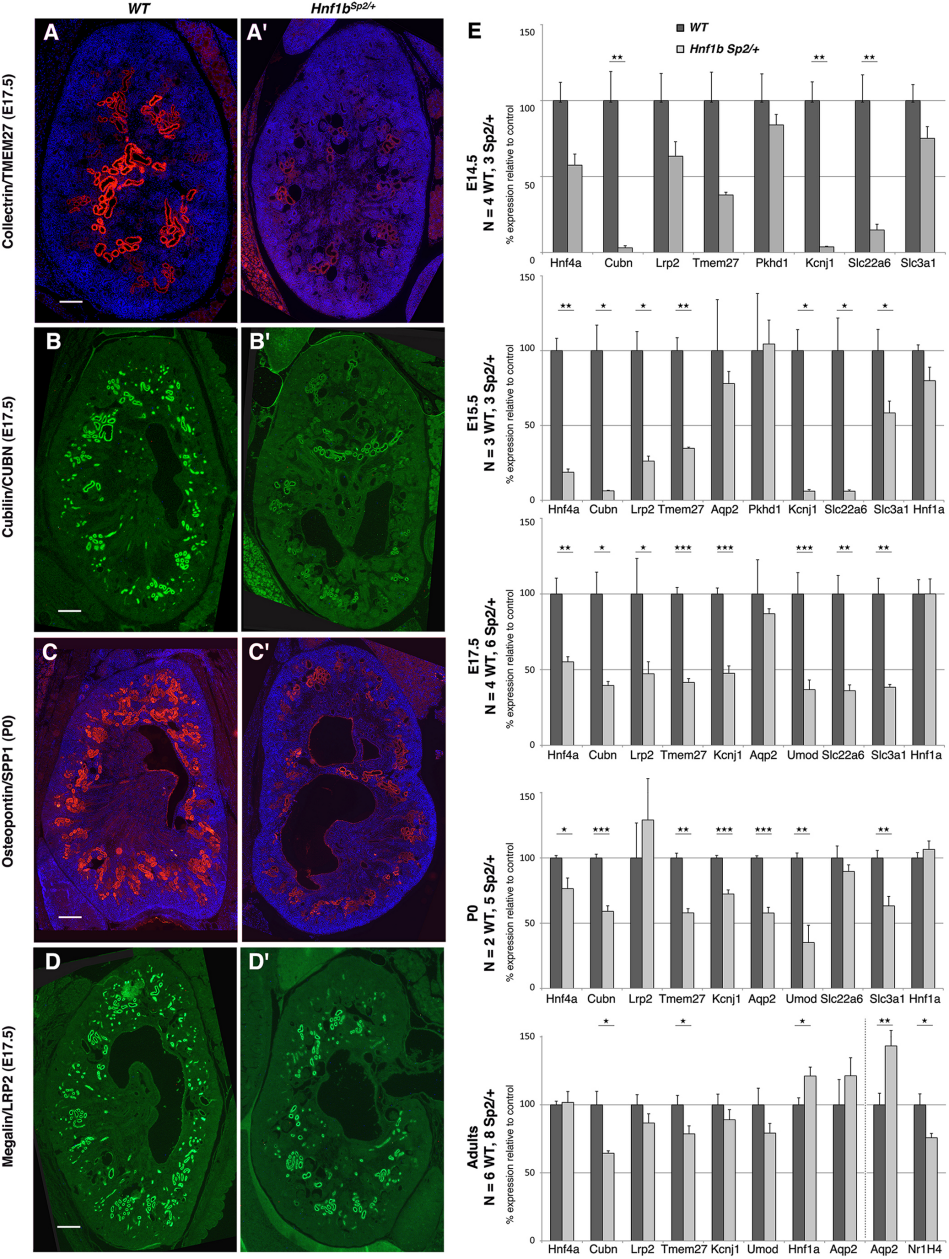
**Reduced expression of a subset of HNF1B targets in *Hnf1b^Sp2/+^* kidneys.** (A-D′) Representative immunostainings of E17.5 WT (A,B,C,D) and *Hnf1b^Sp2/+^* kidneys (A′,B′,C′,D′) show strongly reduced expression in *Hnf1b^Sp2/+^* kidneys of TMEM27, CUBN and SPP1 (A′,B′,C′), whereas LRP2 expression (D′) is moderately reduced. (E) qRT-PCR analysis of selected HNF1B targets at different stages show, consistent with mRNA-seq data, significant and strong downregulation of *Hnf4a*, *Cubn*, *Lrp2*, *Tmem27*, *Kcnj1*, *Umod*, *Slc22a6* and *Slc3a1* during embryo stages up to P0. Also shown is qRT-PCR of adult mice: vertical line separates 3-month-old from two 6-month-old samples. *Cubn *and* Tmem27* remained downregulated in adults, whereas *Aqp2*, downregulated at P0, becomes significantly upregulated in adults (6 months). Note downregulation of the target gene *Nr1h4* in adults (6 months). The numbers (N) of WT and *Hnf1b*^Sp2/+^ samples used at each stage are indicated. Unpaired Student's *t*-test, *P<0.05, **P<0.01 and ***P<0.001. Scale bars: 200 μm.

RNA-seq data were further validated by *in situ* hybridization (ISH), immunostaining and additional qRT-PCR analyses. ISH of *Fbp1* and *Spp2*, two early PT anchor genes (Thiagarajan et al., 2011), revealed a strong downregulation in *Hnf1b^Sp2/+^* kidneys of *Fbp1* transcripts and, to a lesser degree, of *Spp2*, both at E17.5 and P0 (Fig. S7). Likewise, immunofluorescence analysis of E17.5 and P0 *Hnf1b^Sp2/+^* kidneys showed strong reductions in TMEM27, SPP1 and CUBN staining and a more modest decrease in LRP2 (Fig. 7 compare A′,B′,C′,D′ with A,B,C,D, respectively).

qRT-PCR analysis indicated that the early PT markers *Hnf4a*, *Lrp2*, *Cubn* and *Tmem27*, and the loop of Henle markers *Kcnj1* (*RomK*) and *Umod* were preferentially downregulated during development up to P0 (Fig. 7E). Additionally, *Aqp2*, a gene downregulated in developing collecting ducts lacking *Hnf1b* (Desgrange et al., 2017) and upregulated in postnatal kidneys of *Hnf1b*-conditional inactivated medullar renal tubules (Gresh et al., 2004), was also found to be downregulated throughout development and progressively increased in *Hnf1b^Sp2/+^* adult kidneys (Fig. 7E).

Remarkably, the expression of *Hnf1a*, which was expressed later than *Hnf1b* and restricted to developing and adult PTs, was only moderately decreased at E15.5 and remained comparable to WT levels at P0 and in postnatal mutant kidneys (Fig. 7E). Thus, HNF1A, which has the same DNA recognition sequence as HNF1B and binds DNA as homo- or heterodimers with HNF1B, was apparently unable to compensate for reduced HNF1B levels during development. Conversely, *Hnf1a^−/−^* adult mice exhibited a severe dysfunction of PTs together with strongly reduced *Lrp2* and *Cubn* expression, despite the increased expression of *Hnf1b* (Terryn et al., 2016). These observations together suggest distinct and sequential functions of HNF1B and HNF1A in developing versus adult PTs. Consistent with this hypothesis, we found that, in sharp contrast to our *Hnf1b^Sp2/+^* mutants, E16.5-E17 *Hnf1a* null kidneys exhibit normal PT expression of CUBN, LRP2, HNF4A, HNF1B and LTA, and the absence of an embryonic renal phenotype (Fig. S8).

Thus, among the various metanephric kidney developmental processes known to be controlled by HNF1B, the genes participating in early PT differentiation and nephron segment maturation (PT, loop of Henle) are those exhibiting a unique response to HNF1B protein dosage, highlighting a differential dosage sensitivity of HNF1B-activated genes during kidney development. Among these targets, *Hnf4a* has been shown to play an important role in PT maturation and function, by direct regulation of many genes involved in transmembrane transport and metabolic processes (Marable et al., 2020), which are also transcriptionally regulated by *Hnf1b* (Fig. 6; Table S3, File 8). Moreover, our results suggest that a fraction of PT genes, including *Lrp2*, *Cubn* and *Sglt2*, in postnatal life become controlled primarily by HNF1A (Terryn et al., 2016), thus replacing, at least in part, the critical role of HNF1B during embryogenesis.

### Preliminary postnatal characterization of heterozygous *Hnf1b^Sp2/+^* mutant mice

As mentioned above (Table S1B), a fraction of heterozygous mice died between P1 and P25. Otherwise, mice lived more than 1 year, beginning to manifest disease symptoms, albeit with variability, from ∼8-10 months. Further histological analysis of adult *Hnf1b^sp2/+^* mice revealed variability in the severity of the renal phenotype, with increased abnormalities observed in aged males and females**.** They usually exhibited unilateral hydronephrosis, with the other kidney less affected, displaying mainly cortical or medullar glomerular cysts with collapsed or rudimentary capillary tufts (Fig. S9B,C,H,I) as well as microcysts (Fig. S9B,I). Dilated Bowman's spaces were often filled with finely granular proteinaceous material (Fig. S9B′,C′,C″,F′,I′,I″). A rare case (1/48 heterozygous mutants) exhibited bilateral severely affected kidneys: one kidney was highly dysplastic/hypoplastic and the other severely hydronephrotic (Fig. S10H,H′), indicating progressive hydronephrosis with age. Accordingly, some old mice (males or females) developed giant hydronephrosis (Table S5A).

Body weight curve analyses showed ∼20% reduction in weight of heterozygous mice relative to WT, with males and females behaving rather similarly, while kidney weight/body weight ratio of males at different ages did not show significant differences compared to WT littermates (Fig. S10, Table S5B).

Urinary analyses at different ages under basal conditions indicated that although 3-month-old *Hnf1b*^Sp2/+^ mutant mice exhibited normal physiological parameters, 6-month-old mice displayed defective urine-concentrating ability, with polyuria and reduced urine osmolality (Table 1). Reduced urine osmolality was also observed after 22 h of water deprivation (Table S5D). By 12 months, although urine volumes remained higher than in WT, urine osmolality was more modestly and non-significantly decreased (Table 1). A similar increase in 24-h urine output and daily water consumed was observed in three independent groups of 5- to 6-month-old mutant mice, followed by an attenuation of these parameters in 12-month-old mice (Table S5C).

**Table 1.**
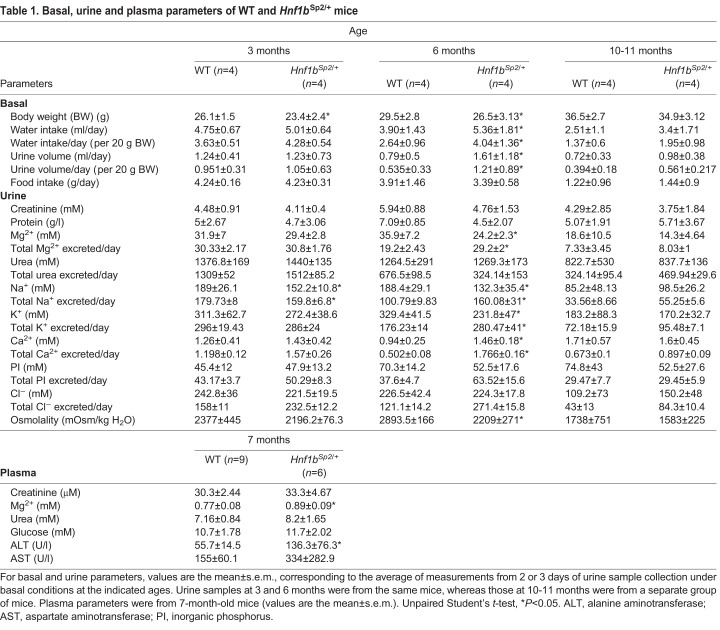
Basal, urine and plasma parameters of WT and *Hnf1b*^Sp2/+^ mice

Consistent with the lower urine osmolality, 6-month-old *Hnf1b*^Sp2/+^ mice had significantly lower urinary Mg^2+^, Na^+^ and K^+^ concentrations, whereas the total excretion of these solutes was increased compared to WT (Table 1), a tendency maintained in 12-month-old mice, although it did not reach significance. Interestingly, urinary calcium concentration was significantly increased despite decreased osmolality. Moreover, plasma analysis in a separate group of mice revealed an increase in creatinine levels, although non-significant, whereas the levels of Mg^2+^ were similar to WT. A significant increase in the levels of alanine aminotransferase (ALT) and a tendency for higher levels of aspartate aminotransferase (AST) were observed, reflecting potential liver dysfunction in heterozygous mutant mice, as reported in some RCAD patients (Iwasaki et al., 1998).

To gain further insight into the pathophysiology of the renal disease, we performed low-molecular-mass urinary proteome analyses from 17 *Hnf1b*^sp2/+^ mutant and 18 WT mice (from 3 months to 17 months of age), using capillary electrophoresis (CE) coupled to mass spectrometry (MS) and tandem mass spectrometry (MS/MS). We identified 40 significant differentially excreted peptides. The most prominent findings associated with our mutants were a substantial decrease in epidermal growth factor (EGF), uromodulin and kidney androgen-regulated protein (KAP), as well as a tendency for an increase in collagen protein fragments in urinary excretion (Table S6). Interestingly, increased collagen peptides and decreased uromodulin levels were also observed in the urinary proteome of a pediatric cohort of RCAD patients (Ricci et al., 2019). Increased excretion of collagen fragments is consistent with the active extracellular matrix (ECM) remodeling that has been related to ECM modifications observed during cyst expansion (Wilson, 2011), while reduced urine uromodulin excretion has been associated with diminished markers of renal tubular function. Particularly, uromodulin, expressed exclusively by the epithelial cells lining the TAL, is excreted into the urine by proteolytic cleavage. The processing and release of uromodulin by TAL cells was found to be regulated by two targets of HNF1B (Ferrè and Igarashi, 2018), the potassium channel *Kcnj1* and the calcium-sensing receptor *Casr*, both expressed in TAL cells (Devuyst et al., 2019). These observations suggest that decreased uromodulin excretion may reflect a global dysfunction of TAL cells, both in our mutants and in RCAD patients.

In conclusion, *Hnf1b*^Sp2/+^ adult mice exhibit progressive renal abnormalities associated with a tendency to defective urinary concentration ability under basal conditions, associated with increased excretion of certain solutes and hypercalciuria, followed by a partial recovery in old animals. Urinary proteome analysis uncovers a particular profile of our heterozygous mutants predictive of progressive decline in kidney function and kidney injury and exhibits common features with the recently reported urine proteome in a pediatric cohort of RCAD (Ricci et al., 2019).

## DISCUSSION

We report here the generation and characterization of a novel mouse model of RCAD disease by reproducing an identified human splicing mutation and show that several of the urogenital defects described in *HNF1B* mutant carriers are replicated at the heterozygous state. These observations confirm that mice are sensitive to haploinsufficiency for *Hnf1b*, further highlighting the importance of precisely replicate human disease mutations.

Although previously described *Hnf1b* mouse mutations (Barbacci et al., 1999; Coffinier et al., 1999) generated a loss-of-function allele similar to our new model, the levels of the HNF1B protein in the heterozygous state were not decreased but rather increased (Kornfeld et al., 2013) (Fig. 1C). The underlying mechanism is not yet identified. By contrast, and as expected, in our heterozygous mutants, the levels of both *Hnf1b* transcript and protein produced by the WT allele were significantly reduced during development. In postnatal life, the levels of HNF1B protein, but not those of transcripts, mainly remained reduced, suggesting that HNF1B activity may additionally be regulated at the post-transcriptional and/or translational level (Fig. 2A,B). Further studies are required to define how the levels or activity of HNF1B protein are regulated. The ubiquitin-proteasome pathway and miRNA-mediated regulation of HNF1B are, among others, possible mechanisms mediating tight regulation of HNF1B protein levels and potentially deregulated in our model. This knowledge will certainly have potential implications for disease therapy.

Although abnormal spliced transcripts are expressed at low levels (Fig. S1B), no evidence of nonsense-mediated mRNA decay (NMD) was observed (data not shown), thus confirming previous studies in human *HNF1B* mutations (Harries et al., 2005). Moreover, we did not detect any of the potential truncated proteins encoded by these alternative spliced transcripts (Fig. S2), and we cannot exclude the fact that truncated proteins were rapidly degraded by the ubiquitin-mediated protein quality control system (Rousseau and Bertolotti, 2018). However, it is more likely that *Hnf1b* mutant spliced transcripts not degraded by NMD are not translated through a process known as nonsense-mediated translational repression (NMTR), a well-documented mechanism described in several frameshift mutations in cancer cells (You et al., 2007) as well as human dominant diseases associated with PTCs (Barefield et al., 2015; Rio Frio et al., 2008). Indeed, these different studies have shown that the products of mutant alleles containing PTCs are degraded or inactivated by two complementary mechanisms, NMD and NMTR, effectively preventing the synthesis of truncated proteins and leading to null alleles and decreased amounts of functional proteins. In summary, these observations suggest that intragenic *HNF1B* mutations leading to PTCs, which represent more than 50% of intragenic mutations (Alvelos et al., 2015), very likely do not lead to the production of truncated proteins, further explaining the observed lack of genotype-phenotype correlations in RCAD patients.

The high variability in the phenotype of *HNF1B* mutant carriers has been explained by diverse mechanisms ranging from modifier genes and environmental factors to interacting cofactors. Our heterozygous mutants did develop heterogeneous urogenital abnormalities with a more severe phenotype in the C57BL/6N background than in the 129/sv background, suggesting that genetic modifiers may indeed either aggravate or attenuate the disease phenotype. Remarkably, even in these two inbred backgrounds, we still observed variability in the phenotype presentation of *Hnf1b^Sp2/+^*, notably at later developmental stages and postnatal life. In particular, in the C57BL/6N background, often one kidney was more severely affected and hydronephrotic than the other. Unilateral affected kidneys have also been reported in RCAD patients (Chen et al., 2010; Heidet et al., 2010). This variability was also observed between *Hnf1b^Sp2/+^* mutants even when they were from the same litter and raised together, in order to reduce environmental exposition variations. One possible mechanism underlying the phenotypic variability of our mutants and *HNF1B* mutant carriers could be the inherent stochasticity in transcription and translation processes (Bar-Even et al., 2006), together with the known increased susceptibility to stochastic delays or interruptions of gene expression due to haploinsufficiency (Johnson et al., 2019).

Global transcriptional profiling indicates that only a subset of the established *Hnf1b* target genes primarily involved in early PT differentiation and onset of nephron tubule mature functions were sensitive to HNF1B reduced levels during development.

The HNF1B target gene *Hnf4a* (Heliot et al., 2013), involved in PT maturation and function (Marable et al., 2020; Martovetsky et al., 2013), is the major transcriptional regulator strongly reduced in our mutants at all developmental stages. Transcriptomic and ChIP analyses have recently shown that HNF4A controls PT maturation via direct activation of transporter and metabolism genes (Marable et al., 2020). A large proportion of HNF4A targets are also targets of HNF1B (Fig. 6C; Table S3, File 8), further suggesting a shared function of HNF1B and HNF4A in developing PTs that relies on synergistic interactions between the two factors at common targets. Such a functional interdependence between HNF1A and HNF4A has already been described in pancreatic islets (Boj et al., 2010) and may explain an increased vulnerability to decreased *Hnf1b* gene dosage. In this context, RNA analysis of renal tissue of a *HNF1B* patient carrying a point mutation showed a strong downregulation of *HNF4A*, *KIF12* and *PPARGC1A* as well as *HNF1B*, whereas other known HNF1 targets remained unchanged (Casemayou et al., 2017). This patient presented a generalized defect in proximal and distal tubular function, incomplete tubular acidosis, hypercalciuria and mitochondrial dysfunction. These observations together highlight the recurrent role of *HNF1B* in PT cells and disease, also in adulthood.

Several of the PT genes strongly downregulated during development in our mutants have additionally been shown to be targets of HNF1A or HNF1A/HNF1B heterodimers in adult kidneys (David-Silva et al., 2013; Kikuchi et al., 2007; Saji et al., 2008). In our mutants, the expression of *Hnf1a* in the PT was only moderately decreased at E17.5 and not affected at P1 or in adults (Fig. 7E; Table S3), suggesting that HNF1A was unable to compensate for decreased HNF1B levels during development. Accordingly, we found normal PT development and marker expression in *Hnf1a* null embryo kidneys (Fig. S8). However, HNF1A appears, at least partially, to compensate in postnatal life, as indicated by the restoration of the expression of some PT markers such as *Lrp2* and *Spp2* in *Hnf1b^Sp2/+^* adult kidneys. Moreover, adult *Hnf1b^Sp2/+^* mice did not show glycosuria, suggesting again that HNF1A replaced HNF1B in the control of *Sglt2* (*Slc5a2*), a gene strongly downregulated in our mutants at both E17.5 and P1. Note however that the renal expression of other known targets, including *Tmem27* and the cationic amino acid exchanger *Slc7a9*, were found to be not affected in *Hnf1a*-deficient mice (Bonzo et al., 2010) and remained downregulated in our heterozygous mutants (Fig. 7; Table S3), highlighting the complexity in the regulatory networks of these two transcription factors in the cells they co-express.

The observed dynamic and temporal regulation patterns (i.e. some genes were strongly downregulated at an early stage but not at a later stage) suggest an increasing complexity in the regulatory network involved in the differentiation and maturation of nephron segments. It is tempting to speculate that similar changes in the promoter occupancy patterns during development involving new recruitments, release and exchange of HNF1A and HNF1B, as described during hepatocyte differentiation (Kyrmizi et al., 2006), also take place during renal development.

Similar to the human disease, glomerular cysts are the most common and earliest feature of the *Hnf1b*^Sp2/+^ embryonic renal phenotype. Although not expressed in glomeruli, HNF1B is expressed in the parietal cells of the Bowman's capsules, suggesting a potential implication in glomerulogenesis. It has been shown that glomerular cysts in *Col4a1* adult mutant mice were preceded by alterations in parietal epithelial cells that abnormally expressed CD44, α-SMA and claudin-1 (Chen et al., 2016). We have not observed such alterations in our mutants. Detailed analysis of glomerular cysts of *Hnf1b^Sp2/+^* embryonic kidneys exhibiting different degrees of Bowman's capsule expansion suggests that glomerular cysts very likely result from delayed and/or abnormal differentiation of early PTs associated with an abnormal glomerulotubular insertion, thus leading to the accumulation of glomerular filtrate and the subsequent expansion of the Bowman's space and glomerular tuft atrophy. Notably, abnormal PT insertion into the Bowman’s capsule was previously described in *Hnf1b*-conditional inactivated mutants in nephron progenitors (Heliot et al., 2013) and more recently further characterized through detailed imaging analyses uncovering, in addition, tubular obstruction (Fiorentino et al., 2020). Neither glomerular cysts nor PT dilatations were observed in *Hnf4a* mutant mice (Marable et al., 2020), thus excluding that the decreased expression of *Hnf4a* in our mutants is related to these phenotypes. These observations are consistent with the earlier and broader role of HNF1B in renal epithelial tubule morphogenesis.

In adulthood, *Hnf1b*^Sp2/+^ mice exhibited defective urinary concentration ability under basal conditions and hypercalciuria until the age of 6 months, followed by a partial restoration by the age of 12 months. Defects in urine concentration have recently been described in mice with specific *Hnf1b* inactivation in the collecting ducts at later stages, a phenotype that was associated with increased AQP2 expression and abnormal apical localization, downregulation of the urea transporter UT-A1 (SlC22A12) and the direct control of Nr1h4 (FXR) (Aboudehen et al., 2017). Although further analyses are required, including urinary concentration ability under different conditions, it is interesting to note that we have also observed a progressive increase in *Aqp2* expression in adult *Hnf1b^Sp2/+^* mice, together with a downregulation of *Nr1h4* (Fig. 7E). *Slc22a12* was also found downregulated at E17.5 and P1 (Table S3).

Adult *Hnf1b*^Sp2/+^ mice also exhibited unilateral hydronephrosis. Physical ureter obstruction, one of the most common causes of hydronephrosis, was not detected in our mutants. Occasionally we have observed dilated ureters at the ureteropelvic junction (Fig. S4, C57BL/6N). Hydronephrosis/hydroureter have been also described in RCAD patients (Adalat et al., 2009) as well as in different mouse models with *Hnf1b* inactivated in the collecting ducts (Aboudehen et al., 2017; Desgrange et al., 2017). Further analyses are required to define whether hydronephrosis is due to the reported polyuria and defective osmoregulation (Aboudehen et al., 2017) or through the previously described perturbations in smooth muscle differentiation of the ureter (Paces-Fessy et al., 2012), or both. Of note, *Hnf4a* mutant mice exhibited at P14 overt non-obstructive hydronephrosis, probably due to the lack of reabsorption in PTs (Marable et al., 2020). It is, therefore, possible that the observed developmental downregulation of *Hnf4a* contributes, at least in part, to the onset of this phenotype.

Metabolic profiling of different organs, including kidney, pancreas and liver, as well as plasma of adult *Hnf1*^Sp2/+^ mice, was previously reported (Torell et al., 2018). We found evidence of impaired amino acid renal metabolism and reduced plasma levels of total free amino acids and increased myo-inositol, which are metabolic parameters reflecting impaired renal function, together with disturbed hepatic metabolism. Likewise, and as reported in RCAD mutant carriers, we observed in adult mutants a significant increase in plasma levels of ALT and a tendency for higher levels of AST, reflecting liver dysfunction (Clissold et al., 2015). Unlike humans, adult mice did not exhibit hypo-magnesemia and/or hyper-magnesuria reported in more than 30% of *HNF1B* adult mutant patients. These results may reflect differences between mouse and humans in ion-transport regulation and/or adaptive mechanisms.

Notably, urinary proteome analysis uncovered a particular profile in our heterozygous mutants, with a substantial decrease in uromodulin and EGF peptides, predictive of progressive decline in kidney function, as well as increased collagen fragments, consistent with excessive ECM turnover. Urinary proteome analysis in a pediatric cohort of RCAD patients revealed a similar signature, with a majority of collagen type I or type III peptide fragments enriched in the urine of RCAD patients and a decrease in uromodulin fragments, together with other additional deregulated peptides (Ricci et al., 2019). Altogether, these observations emphasize a more global implication of HNF1B in postnatal kidney function in our mutants, involving different nephron segments and collecting ducts and likely associated with a deregulation of additional identified target genes (reviewed by Shao et al., 2020). Consistent with a role of HNF1B in postnatal tubular maintenance and transport activity, recent clinical data of a pediatric cohort of *HNF1B* mutant carriers show that abnormal tubular electrolyte handling develops progressively with age (Adalat et al., 2019).

Thus, our study shows how constitutive heterozygous germline mutations affect early kidney development and provides further insights into the mechanisms underlying the renal developmental abnormalities associated with RCAD and their consequences in postnatal life. In summary, the *Hnf1b*^Sp2/+^ mouse model represents a unique clinical/pathological viable model of the human disease and promises to be important in the integrative evaluation, in the context of the whole animal, of the broad facets of this disease, ranging from various developmental abnormalities to kidney, pancreas and liver dysfunctions as well as tumorigenesis (Yu et al., 2015).

## MATERIALS AND METHODS

### Generation of a mouse model carrying a point mutation at the intron-2 splice donor site

The knock-in mouse carrying a human splicing point mutation was generated according to a proposal of S.C. with the support of the GIS-Institut des Maladies Rares and the Mouse Clinical Institute. The WT sequence GAC/**g** taagtgttttaacctt was mutated to GAC/**t** taagtgttttaa**g**ctt sequence (upper-case letters show end exon-2 bases, lower-case letters indicate the intron-2–exon-2 junction sequence, bold text indicates the point mutations, underlined text indicates the HindIII restriction site). The splice mutation into the *Hnf1b* locus was introduced by homologous recombination into 129/sv embryonic stem (ES) cells. Two correctly recombined ES clones were obtained, which were additionally used to differentiate into embryoid bodies and confirm the generation of expected *Hnf1b* spliced transcripts before the generation of the mouse line. We additionally performed mRNA analysis of *Hnf1b^Sp2/+^* ES cells differentiated into embryoid bodies in the presence of the NMD inhibitor cycloheximide (100 µg/ml, 5 h) as described (Barbacci et al., 1999) and found no alterations in the levels of alternative spliced transcripts (data not shown). Chimeric mice were obtained by microinjection into C57BL/6N blastocysts. The *LoxP*-flanked neomycin-resistance cassette was located within intron-1 and subsequently excised by breeding heterozygous mutant mice with a ‘Cre deleter’ mouse line. Thus, the mutated allele encompassed the point mutation at the splice donor site in addition to a single LoxP site and HindIII restriction site aagctt within intron-2. Mice homozygous for the splicing mutation were lethal before gastrulation. The line was maintained as heterozygous either in a mixed C57BL/6N x129sv or in pure C57BL/6N and 129/sv backgrounds, in general by crossing heterozygous males with WT females. Whatever the background, we noticed that the relative severity of the renal phenotype of heterozygous mutants was higher in the descendants of crosses of heterozygous males and females. In addition to the renal phenotype, mice in the C57BL/6N background exhibited unilateral or bilateral absence of eyes (30% of *n*=36 *Hnf1b^Sp2/+^* males and *n*=20 *Hnf1b^Sp2/+^* females) manifested from embryo stages. Intriguingly, the same eye phenotype was observed in ∼15% of heterozygous for a null allele [see the European Mouse Mutant Archive (EMMA; http://www.emmanet.org) description]. This ocular phenotype remains undefined. It may be linked to the Rd8 mutation of the *Crb1* gene present in the strain C57BL/6N (Mattapallil et al., 2012). However, there is no evidence that it is related to HNF1B dosage, because it is present in the two heterozygous *Hnf1b^Sp2/+^* and *Hnf1b^lacZ^*^/+^ mutants.

Mice heterozygous for the *Hnf1b* null allele (*Hnf1b^lacZ^*^/+^; international designation *Hnf1b*^tm1Sce^), with the *lacZ* gene and the SV40 polyadenylation sequence and neomycin resistance cassette, replacing the first exon of *Hnf1b* (Barbacci et al., 1999), were maintained as heterozygotes. The mouse line in pure 129/sv (129/sv-*Hnf1b*^tm1Sce^) and C57BL/6N (C57BL/6N-*Hnf1b*^tm1Sce^) backgrounds are available at EMMA (EMMA IDs EM:07817 and EM:07827, respectively) together with the phenotype description. The *Hnf1a**^+^**^/−^* mice were provided by Frank Gonzalez (Laboratory of Metabolism, National Cancer Institute/Center for Cancer Research) (Lee et al., 1998) and maintained as heterozygotes in the C57BL/6N background.

Animal care and the experimental protocols were approved by and conducted in accordance with French and European ethical legal guidelines and the local ethical committee for animal care (Comité d'éthique en Expérimentation Animale Charles Darwin number 5, approval number 04817.02), respecting the 3R rules.

Paraffin-embedded human fetal tissues were obtained from family members with informed consent approved by the Ethics committee of Debré Hospital (Anne-Lise Delezoide, Service de Foetopathologie, Hôpital Robert Debré, Paris, France), as previously reported in Haumaitre et al. (2006) and according to the principles expressed in the Declaration of Helsinki.

### ISH and immunohistochemistry

ISH on paraffin sections was performed as described (Lokmane et al., 2008). The *Fbp* and *Spp2* cRNA probes were generated by PCR (GUDMAP database; https://www.gudmap.org). Embryos and postnatal kidneys up to 2 months of age were fixed with 60% ethanol/11% formaldehyde and 10% acetic acid. Adult kidneys (>2 months) were fixed in alcoholic Bouin (Duboscq-Brasil) solution and paraffin sections were used for H&E histological analysis and immunohistochemistry. Antibody staining on paraffin sections was performed as described (Desgrange et al., 2017; Lokmane et al., 2010). For each probe (ISH) or antibody, sections from at least three different embryos were used. The primary and secondary antibodies are listed in Table S7.

### RNA extraction and RT-PCR

Both kidneys of each embryo up to P1 were dissected in ice-cold Dulbecco’s modified Eagle medium (DMEM) and washed in ice-cold PBS. WT and heterozygous mutant embryos were from the same litter. Adult kidneys were cut sagittal: one-half was used for histology, one-quarter for RNA extraction and the other quarter for protein extraction. Total RNA was extracted using an miRNeasy Mini Kit (Qiagen). Samples were treated with Dnase1 on the columns according to the manufacturer's instructions, and 250-500 ng was reverse transcribed using a High-Capacity cDNA Reverse Transcription Kit (Applied Biosystems). RT-PCR was performed using Fast SYBR Green Master Mix (Applied Biosystems) and the Step-One Plus system (Applied Biosystems), as described (Paces-Fessy et al., 2012). The primers used are listed in Table S2. Number of kidney samples is indicated in figure legends. The mean and s.e.m. were calculated by genotypes, and the statistical significance was determined using unpaired Student's *t*-test, with *P*<0.05 considered significant.

### Semiquantitative RT-PCR

Total RNA from microdissected kidneys was extracted and subjected to semiquantitative RT-PCR as described (Lokmane et al., 2008) with the following modifications. The conditions were chosen so the RNAs analyzed were in the exponential phase of amplification by performing different PCR cycles as indicated in Fig. S1. PCR products were resolved in 2% agarose/TBE ethidium bromide gels and photographed using a GELDOC documentation system. Densitometry quantification was performed with ImageJ software. Primer sequences used were *Gapdh* for normalization and, for *Hnf1b*, vATG and v695 (Table S2).

The cDNA sequences of spliced isoforms and the encoded truncated proteins from the mutated *Hnf1b* allele are listed below:

#### Spliced isoform A/B **Δ**32 bp exon-2

##### ▪ cDNA sequence (underlined text shows retained exon-2 minus 32 bp):

ATGGTGTCCAAGCTCACGTCGCTCCAGCAAGAACTCCTGAGTGCCCTGCTGAGCTCCGGAGTCACCAAGGAAGTGCTGATCCAGGCCTTGGAGGAGTTACTGCCGTCCCCGAATTTCGGGGTGAAGCTGGAGACACTGCCCCTGTCCCCCGGGAGCGGGGCGGATCTCGACACCAAGCCGGTTTTCCATACTCTCACCAATGGCCACGCCAAGGGCCGCTTGTCTGGGGACGAGGGCTCAGAGGACGGCGACGACTATGACACTCCTCCCATCCTCAAAGAGCTCCAGGCGCTCAACACCGAGGAGGCCGCGGAGCAGCGGGCCGAGGTGGACCGGATGCTCAGCGAGGACCCGTGGAGGGCTGCCAAAATGATCAAGGGATACATGCAACAGCACAATATCCCCCAGAGGGAGGTGGTCGATGTCACAGGCCTGAACCAATCCCACCTCTCTCAACACCTCAACAAGGGCACCCCCATGAAGACCCAGAAGAGAGCTGCCCTGTACACTTGAg

##### ▪ Putative encoded protein (170 amino acids; bold text shows WT amino acid sequence, the asterisk indicates the STOP codon):

MVSKLTSLQQELLSALLSSGVTKEVLIQALEELLPSPNFGVKLETLPLSPGSGADLDTKPVFHTLTNGHAKGRLSGDEGSEDGDDYDTPPILKELQALNTEEAAEQRAEVDRMLSEDPWRAAKMIKGYMQQHNIPQREVVDVTGLNQSHLSQHLNKGTPMKTQKRAALYT*VQPD

#### Spliced isoform A **Δ**exon-2

##### ▪ cDNA sequence (underlined text shows spliced exon-3):

ATGGTGTCCAAGCTCACGTCGCTCCAGCAAGAACTCCTGAGTGCCCTGCTGAGCTCCGGAGTCACCAAGGAAGTGCTGATCCAGGCCTTGGAGGAGTTACTGCCGTCCCCGAATTTCGGGGTGAAGCTGGAGACACTGCCCCTGTCCCCCGGGAGCGGGGCGGATCTCGACACCAAGCCGGTTTTCCATACTCTCACCAATGGCCACGCCAAGGGCCGCTTGTCTGGGGACGAGGGCTCAGAGGACGGCGACGACTATGACACTCCTCCCATCCTCAAAGAGCTCCAGGCGCTCAACACCGAGGAGGCCGCGGAGCAGCGGGCCGAGGTGGACCGGATGCTCAGAGTTCAACCAGACAGTCCAGAGCTCTGGAAACATGACAGACAAAAGCAGTCAGGATCAGCTGCTGTTTCTCTTTCCAGAGTTCAGTCAACAGAACCAGGGGCCTGGGCAGTCGGAGGACACCTGCTCCGAGCCCACCAACAAGAAGATGCGCCGCAACCGGTT**TAA**a

##### ▪ Putative encoded protein (169 amino acids; bold text shows WT amino acid sequence, the asterisk indicates the STOP codon):

**MVSKLTSLQQELLSALLSSGVTKEVLIQALEELLPSPNFGVKLETLPLSPGSGADLDTKPVFHTLTNGHAKGRLSGDEGSEDGDDYDTPPILKELQALNTEEAAEQRAEVDRML**RVQPDSPELWKHDRQKQSGSAAVSLSRVQSTEPGAWAVGGHLLRAHQQEDAPQPV*

#### Spliced isoform B **Δ**exon-2

##### ▪ cDNA sequence (underlined text shows spliced exon-3, bold text shows the STOP codon sequence):

ATGGTGTCCAAGCTCACGTCGCTCCAGCAAGAACTCCTGAGTGCCCTGCTGAGCTCCGGAGTCACCAAGGAAGTGCTGATCCAGGCCTTGGAGGAGTTACTGCCGTCCCCGAATTTCGGGGTGAAGCTGGAGACACTGCCCCTGTCCCCCGGGAGCGGGGCGGATCTCGACACCAAGCCGGTTTTCCATACTCTCACCAATGGCCACGCCAAGGGCCGCTTGTCTGGGGACGAGGGCTCAGAGGACGGCGACGACTATGACACTCCTCCCATCCTCAAAGAGCTCCAGGCGCTCAACACCGAGGAGGCCGCGGAGCAGCGGGCCGAGGTGGACCGGATGCTCAGAGTTCAGTCAACAGAACCAGGGGCCTGGGCAGTCGGAGGACACCTGCTCCGAGCCCACCAACAAGAAGATGCGCCGCAACCGGTT**TAA**a

##### ▪ Putative encoded protein (143 amino acids; bold text shows WT amino acid sequence, the asterisk indicates the STOP codon):

**MVSKLTSLQQELLSALLSSGVTKEVLIQALEELLPSPNFGVKLETLPLSPGSGADLDTKPVFHTLTNGHAKGRLSGDEGSEDGDDYDTPPILKELQALNTEEAAEQRAEVDRML**RVQSTEPGAWAVGGHLLRAHQQEDAPQPV*

Note that the STOP codons of putative encoded proteins are followed by a purine, which is predicted to positively influence translational termination efficiency [UAAR, UAGR, UGAR (R, purine) (McCaughan et al., 1995)].

#### *Hnf1b* mouse exon-2

CGAGGACCCGTGGAGGGCTGCCAAAATGATCAAGGGATACATGCAACAGCACAATATCCCCCAGAGGGAGGTGGTCGATGTCACAGGCCTGAACCAATCCCACCTCTCTCAACACCTCAACAAGGGCACCCCCATGAAGACCCAGAAGAGAGCTGCCCTGTACACTTGGTACGTCAGAAAGCAACGGGAGATCCTCCGAC

Underlined text shows the 32 bp of exon-2 spliced out of mouse exon-2.

### Western blots from embryo/adult kidneys

Human embryonic kidney (HEK) 293 cells were maintained and transiently transfected as described (Barbacci et al., 2004), with either expression vectors of full-length HNF1B or the truncated spliced isoforms [A Δexon-2, 169 amino acids; B Δexon-2, 143 amino acids; A/B Δ32 bp, 170 amino acids cloned into the pCB6 vector (Barbacci et al., 2004)].

Transfected cells were washed with ice-cold PBS plus Roche protease inhibitors, scraped, transferred into Eppendorf tubes and centrifuged for 5 min at 1006 ***g***. The cellular pellet was resuspended into ice-cold HNB buffer [0.5 M sucrose, 10 mM Tris-HCl pH 7.4, 60 mM KCl, 0.5 mM spermidine, 0.15 mM spermine, 1 mM DTT plus complete protease inhibitor cocktail (Roche)], frozen and thawed four times in liquid N_2_, and centrifuged for 10 min at 11,180 ***g***. The supernatant containing the whole-cell extracts was frozen in liquid N_2_ and kept at −80°C.

The two kidneys of each embryo of at least three (*n*=3) different litters were pooled, frozen in liquid N_2_ and lysed in ice-cold lysis buffer using 23 g and 26 g syringes. Adult kidney pieces were frozen in liquid N_2_, reduced to a fine powder under liquid N_2_ and further lysed in lysis buffer using a syringe. Lysis buffer contained 15% glycerol, 10 mM Tris-HCl pH 7.4, 150 mM NaCl, 5 mM EDTA, 1% NP40, 3 mM sodium pyrophosphate and 50 mM sodium fluoride, with complete protease inhibitor cocktail (Roche) added before using. Samples were centrifuged for 15 min at 18,894 ***g*** at 4°C, and the supernatant was frozen in liquid N_2_ and kept at 80°C. Protein concentration was determined using a Pierce™ BCA Protein Assay kit. Whole-cell extracts containing 20-30 μg of protein were prepared in SDS sample buffer and subjected to SDS-PAGE (4-15% Mini-PROTEAN^®^ TGX™ Precast Protein Gels, Bio-Rad). After the proteins were transferred onto a 0.2 µm nitrocellulose membrane (Bio-Rad) and blocked with TBST (50 mM Tris-HCl pH 7.4, 150 mM NaCl, 0.1% Tween, 5% skim milk), immunoblotting was performed by overnight incubation in TBST 1% skim milk buffer with a rabbit polyclonal antibody against HNF1B (1:500) previously validated (Haumaitre et al., 2006) raised in the laboratory against residues 39-89 of the mouse HNF1B protein, as well as an HNF1B antibody (1:1000; Sigma-Aldrich) raised against residues 23-120 of the human HNF1B protein. Secondary horseradish peroxidase-conjugated antibodies (goat anti-rabbit; Santa Cruz Biotechnology) were incubated at room temperature in blocking buffer for 2 h. After visualization, blots were stripped and incubated with mouse monoclonal α-tubulin antibody (Sigma-Aldrich) at 1:10,000 used as a loading control and then exposed to secondary antibody as above (antibodies are listed in Table S7). Positive HNF1B bands were detected by chemiluminescence (Super Signal TM West Femto, ThermoFisher Scientific). Western lightning Plus-ECL Perkin Elmer was used to detect α-tubulin. Images were captured with G-BOX Syngene Europe, Chemiluminescence Image Capture software and quantified by Image J. WT and HNF1B proteins normalized by α-tubulin expression were quantified from each litter. A WT sample was assigned a 100% value and the other samples of the same litter WT and heterozygotes were referred to as a percentage of this value. This allowed the comparison of different litters of a given embryonic stage. Statistical significance was determined using unpaired Student's *t*-test, using Prism 6.00 (GraphPad Software, San Diego, CA, USA). *P*<0.05 was considered significant.

### Plasma and urine analyses

Urine and plasma were obtained from age- and sex-matched heterozygous and WT mice (males). They were housed in a light- and temperature-controlled room with *ad libitum* access to tap water and standard chow (Diet AO4, SAFE, France). Twenty-four-hour urine samples collected under mineral oil to avoid evaporation were obtained at baseline in individual metabolic cages, after 2-3 days habituation. Each 24 h, animals were weighed and food intake, water intake, urine volume and fecal weight recorded. Blood was sampled by retro-ocular puncture, in general 2 weeks after being in the metabolic cages, and plasma samples were kept at −80°C. The urinary concentration ability was tested after 22 h of water deprivation. Urinary creatinine, urea and electrolytes, plasma urea, creatinine, Mg^2+^, AST and ALT were measured on an Olympus AU400 Chemistry Analyzer (ICB-IFR2, Laboratoire de Biochimie UFR de Médecine, Paris, France). Osmolality was measured using a vapor pressure osmometer (Wescor 5500, USA).

### Statistical analysis

Data are presented as mean±s.e.m. Unpaired Student’s *t*-test or unpaired Student's *t*-test with Welch correction were used for statistical analysis. *P*<0.05 was considered significant.

### Urinary proteome analysis

Urine samples were obtained from 17 *Hnf1b^Sp2/+^* and 18 WT mice from the age of 3, 8, 12 and 17 months. Urine from WT and mutant mice (males) was collected by spontaneous voiding or after animals were placed individually in metabolic cages as described. The total volume of 24-h urine was aliquoted and frozen at −80°C. A 150-µl sample of mouse urine was diluted with the same volume of urea buffer (2 M urea, 10 mM NH_4_OH, 0.2% SDS). Then, 150 µl of the urine samples was ultrafiltrated, desalted, lyophilized and resuspended for proteome analysis as described (von zur Muhlen et al., 2012).

### CE-MS analysis, data processing and statistical analysis

CE-MS analysis was performed using a Beckman Coulter Proteome Lab PA800 capillary electrophoresis system (Fullerton, CA, USA) online coupled to a micrOTOF II MS (Bruker Daltonic, Bremen, Germany), as described (Coon et al., 2008; Mischak et al., 2013). For normalization caused by analytical variances and differences in urine dilution, MS signal intensities were normalized relative to 41 internal standard peptides generally present in at least 90% of all mouse urine samples, with small relative s.d. (von zur Muhlen et al., 2012). The peak lists obtained characterized each peptide by its molecular mass (in Da), normalized CE migration time (in min) and normalized signal intensity. The data of all detected peptides were deposited, matched and annotated in a Microsoft SQL database as previously described, allowing further analysis and comparison of multiple samples (Siwy et al., 2011). The main differences in peptides between the WT and *Hnf1^Sp2/+^* urine were obtained using the *P*-values based on Wilcoxon rank-sum test. Statistical adjustment of *P*-values due to multiple testing was performed by the Benjamini and Hochberg method. Peptides that were detectable in >90% of mice and reached an adjusted *P*-value of <0.05 were further considered as relevant.

### Transcriptional profiling at different stages by mRNA-seq

The two kidneys from heterozygous *Hnf1b*^Sp2/+^ mutants and WT embryos in the C57BL/6N background were microdissected from the same litter. This requirement limited the number of samples used in general to two WTs and two heterozygous mutants, independently of the sex. Note that we found a similar phenotype in males and females. The stages analyzed included E14.5 (when the mutant kidneys were morphologically normal and considered as pre-disease) and at disease stages E15.5, E17.5 and P1. Note that at E15.5 we performed deep sequencing from pooled samples of three WT and three *Hnf1b^Sp2/+^* (six kidneys each sample). RNA-seq of E14.5, E15.5 and P1 samples was performed at the Alexander Fleming Institute, Genomics Facility, Greece; RNA-seq of E17.5 samples was performed at Fasteris, Switzerland (Table S8).

RNA from microdissected kidneys was extracted by Tryzol, using a Qiagen miRNA mini kit for the extraction of total RNA and miRNAs. The quality of the RNA samples was assessed on an Agilent Bioanalyzer system using an RNA 6000 Nano Kit (Agilent Technologies) and RNA with a Ring higher than 8 was used. Then, 1-2 μg of total RNA was used for mRNA isolation using a Dynabeads^®^ mRNA DIRECT™ Micro Kit (ThermoFisher Scientific). mRNA was digested with RNase III, purified, hybridized and ligated to Ion Adaptors (ThermoFisher Scientific), reverse transcribed, barcoded and amplified, using an Ion Total RNA-Seq Kit v2 (ThermoFisher Scientific). RNA-seq was performed on an Ion Proton™ System (ThermoFisher Scientific), according to the manufacturer's instructions. The prepared libraries were quantified and pooled together in duplicates at the required concentration. The pools were then processed on a OneTouch 2 instrument and enriched on a OneTouch ES station. Templating was performed using an Ion PI™ Template OT2 200 Kit (ThermoFisher Scientific) and sequencing with an Ion PI™Sequencing 200 Kit on Ion Proton PI™ chips (ThermoFisher Scientific) according to commercial protocols. The resulting RNA-seq BAM files were analyzed with the Bioconductor package metaseqR (Moulos and Hatzis, 2015), applying the edgeR methodology for differential expression analysis with default settings (http://www.bioconductor.org/packages/release/bioc/html/edgeR.html).

High-throughput DNA sequencing using Illumina technology consisted of processing *in vitro* samples to generate a library of short inserts (the DNA Colonies Template Library). The library was sequenced on an Illumina HiSeq 2000. For each lane, 130-150 million DNA colonies producing pass filter sequences were assured. The read lengths are 1×50 bp or 1×100 bp for single-read runs using the forward sequencing primer. The inserts can also be sequenced from both ends using a ‘forward’ and a ‘reverse’ sequencing primer, generating paired reads of 2×100 bp. The data were processed using bioinformatics tools to extract biologically useful information. To homogenize mRNA-seq comparisons, RNA-seq files were all analyzed as described above (Moulos and Hatzis, 2015).

## Supplementary Material

Supplementary information
