# Supplementary material for: Hnf1b haploinsufficiency differentially affects developmental target genes in a new renal cysts and diabetes mouse model
Source: Dis Model Mech. 2021 May 4;14(5):dmm047498. doi: 10.1242/dmm.047498 (PMC8126479; doi:10.1242/dmm.047498)
Supplement: Supplementary information [file dmm-14-047498-s1.pdf]

Figure S1: **Structure and generation of the *Hnf1b* intron-2 spliced mutant mice.** **A:** Genomic organization of the *Hnf1b* locus, illustrating the 9 coding exons and main functional domains (N-terminal Dimerization, POU-specific (POU_S) and POU-homeodomain (POU_H) and C-terminal transactivation domain). Also indicated are the mutation G to T introduced at the intron-2 splice donor site; the Nuclear Localization Signal (NLS) and the location of the alternative exon of 78 base pairs (bp) present in the *Hnf1b isoform A* (variant A) or without (variant B). **B**: Characterization of abnormal spliced *Hnf1b* transcripts generated by the *HNF1b$^{Sp2/+}$* mutant allele. Representative semiquantitative RT-PCR from P1- kidney RNA of *WT* (lanes a, b) and *HNF1b$^{Sp2/+}$* (lanes c, d), using primers located in exon 1 and exon 3 (Material and Methods). Amplifications were performed for different cycles ranging 27 to 36 for *Hnf1b* (lanes a, c and lanes b, d correspond respectively to 30 and 33 cycles) and for *Gapdh*, used to normalize for RNA amount for 24 to 30 cycles, (e, f, show 28 cycles). The structure of spliced products is depicted on the right panel. Sequence of PCR products indicated that *WT* express *Hnf1b* variants A and B, while *Hnf1b$^{Sp2/+}$* mice express four additional abnormal spliced transcripts corresponding to variants A and B lacking either exon 2 or the last 32bp of exon2 because production of the cryptic splicing site within exon 2 (ss). Quantification of transcript levels normalized by *Gapdh,* in P1 *HNF1b$^{Sp2/+}$* relative to *WT*, were for the normal transcripts *Hnf1b* (variant A+B) 67% and for the spliced isoforms (A Δexon2 + B Δexon2) 15% and for A+B Δ32pb 14%. The cDNA sequence of spliced isoforms and the encoded truncated proteins from the mutated *Hnf1b* allele are shown in Material and Methods **C**: Cryptic splice site (SS) within exon-2 of mouse *Hnf1b* and human *HNF1B* gene and the 5'splice consensus sequence. Italics show part of the sequence of exon2 spliced out. **D** Semiquantitative RT-PCR from the indicated embryo stages and adult kidney RNA of *WT* and *HNF1b$^{Sp2/+}$* show that heterozygous mutants exhibit a similar pattern of alternative spliced variants to those shown in Panel B at P1.

Disease Models & Mechanisms • Supplementary information

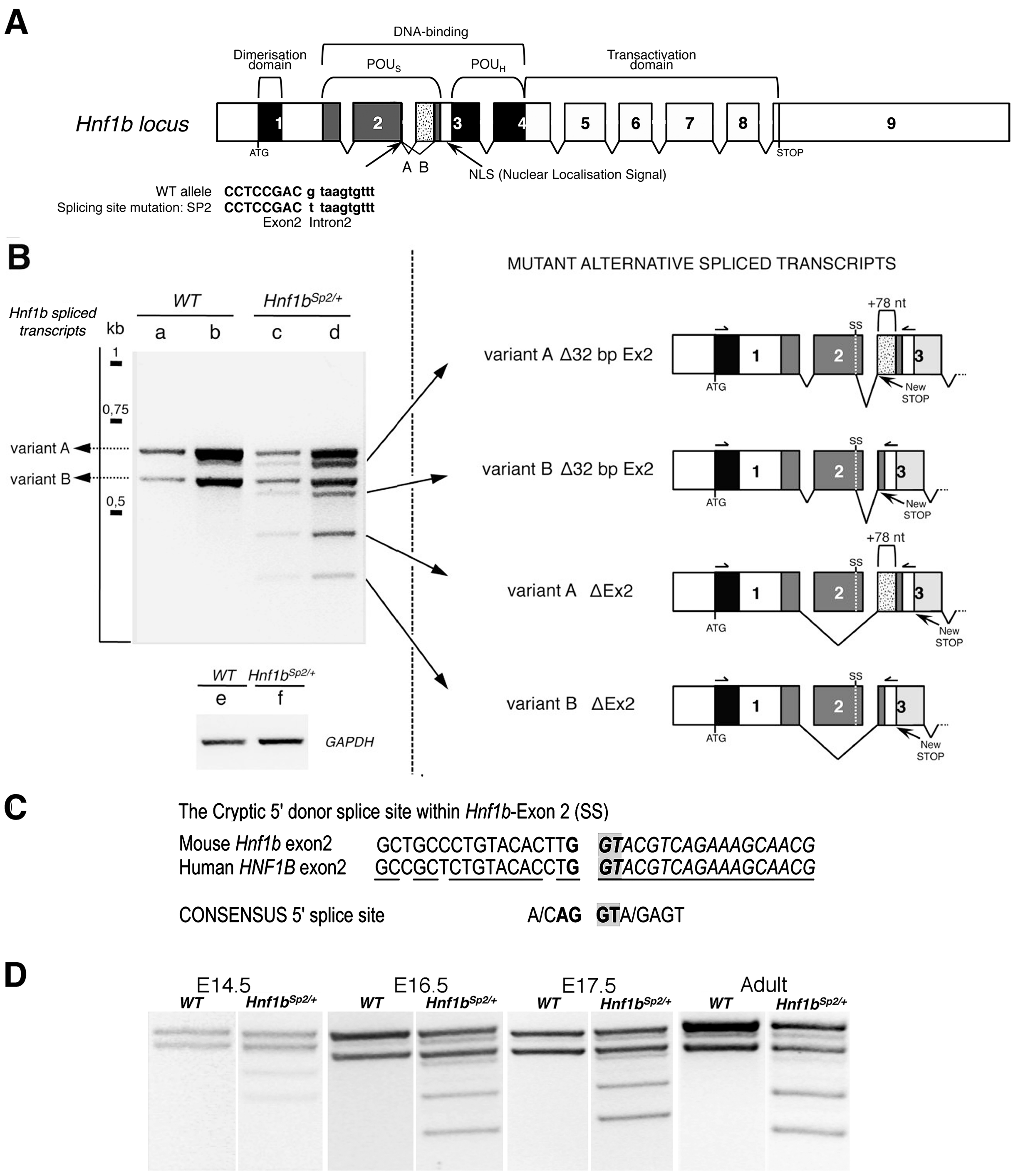

Figure S1

Disease Models & Mechanisms • Supplementary information

Figure S2: Representative western blots of *WT* and *Hnf1b*$^{Sp2/+}$ kidney extracts at the indicated stages and adult mice. Note that HNF1B isoforms A and B migrate very close and were quantified together. α-tubulin was used to normalize for protein amount as described in Material and Methods. A-C, E: controls corresponding to extracts from transfected cells with expression vectors for HNF1B-variant A (557 amino acids) and for truncated spliced mutant isoforms A Δexon2 (169 amino acids), B Δexon2 isoform (143 amino acids) or A/B Δ32pb (170 amino acids). C: extracts from transfected cells with HNF1B-A, the isoforms A/BΔ32pb, AΔexon2 or BΔexon2 as controls and the indicated increased amounts of E17.5 whole kidney extract from *Hnf1b*$^{Sp2/+}$ showing the absence of truncated isoforms. D shows extracts PO and P1 extracts from respectively the same litters. Dotted vertical line in Panel D (P1), denote that a lane was cut out because of a bubble during transfer, making no possible quantification analysis; otherwise P1 samples were all from the same gel.

Disease Models & Mechanisms • Supplementary information

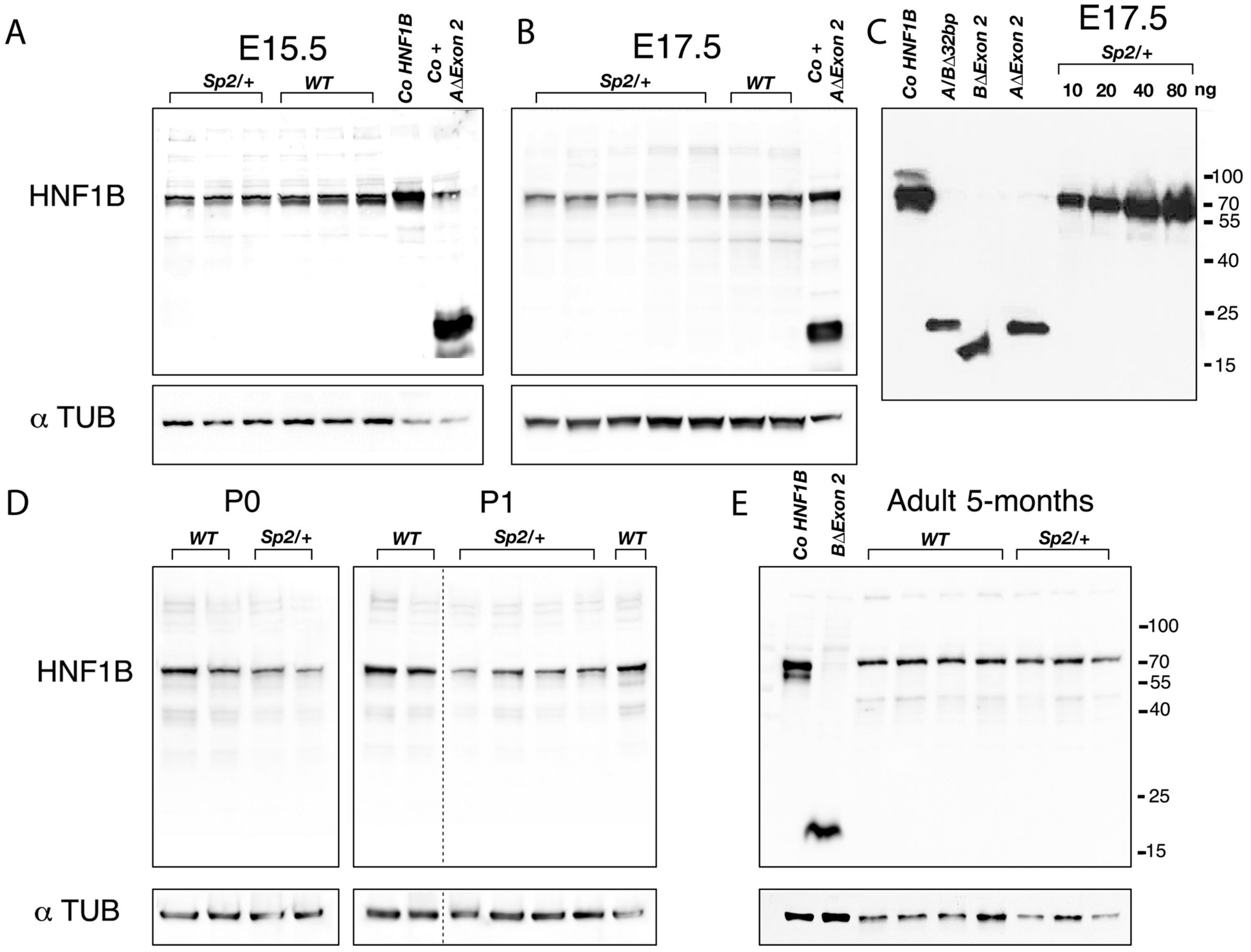

Figure S2

Disease Models & Mechanisms • Supplementary information

Figure S3: *Hnf1b*$^{Sp2/+}$ E14.5 embryo kidneys show normal histology, branching morphogenesis and nascent nephron structures

A) **Hematoxylin and Eosin (H&E) stained sections:** Upper panel**,** F1 background (mixed C57BL/6Nx129/sv background), middle panel C57BL/6N and Lower panel 129/sv inbred backgrounds, showing in each case one E14.5 *WT* kidney and the two *Hnf1b*$^{Sp2/+}$ kidneys of the same litter. Scale bar: 200µm.

B) **Immunostainings** of **Calbindin and PAX-2 show normal expression pattern in E14.5 *Hnf1b*$^{sp2/+}$ kidneys.** Calbindin-D-28K expressed in the emerging ureteric bud branches, highlights branching morphogenesis, while PAX-2 denotes induced metanephric mesenchyme, UB branches, nascent nephrons, renal vesicles (RV) and S-shaped bodies (SSB) (magnifications in insets)

C) HNF1B and WT1 immunostaining analysis on E14.5 serial kidney sections show rare examples at this stage of expanded Bowman's capsule (g) and cystic tubules (cy) in *Hnf1b*$^{Sp2/+}$ (upper panels). Pax2 immunostainings at different stages (E16.5, E18.5) show similar pattern in WT and *Hnf1b*$^{Sp2/+}$ (middle panels), indicating that the expression of Pax2 in *Hnf1b*$^{Sp2/+}$ is not affected throughout development. Note, however at E17.5 the presence of glomerular cysts and medullar tubular dilatations, likely Loop of Henle, which are not stained by Pax2. **Lower panel illustrate a rather similar expression profile of HNF1A in PTs of E17.5 WT and *Hnf1b*$^{Sp2/+}$**, in contrast to the observed decreased expression of HNF1A at earlier stages (see Figure 3).

Disease Models & Mechanisms • Supplementary information

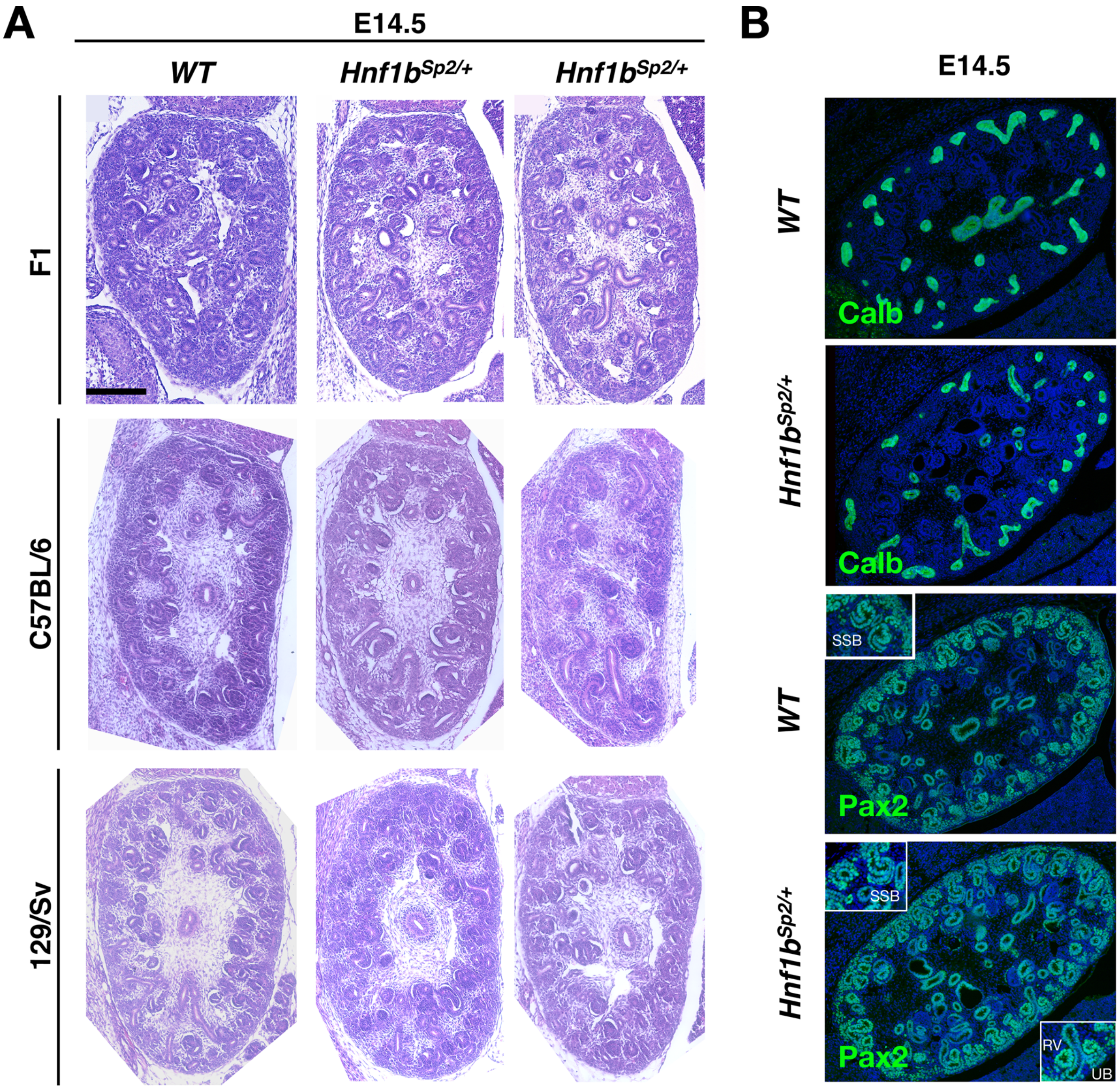

Figure S3

Disease Models & Mechanisms • Supplementary information

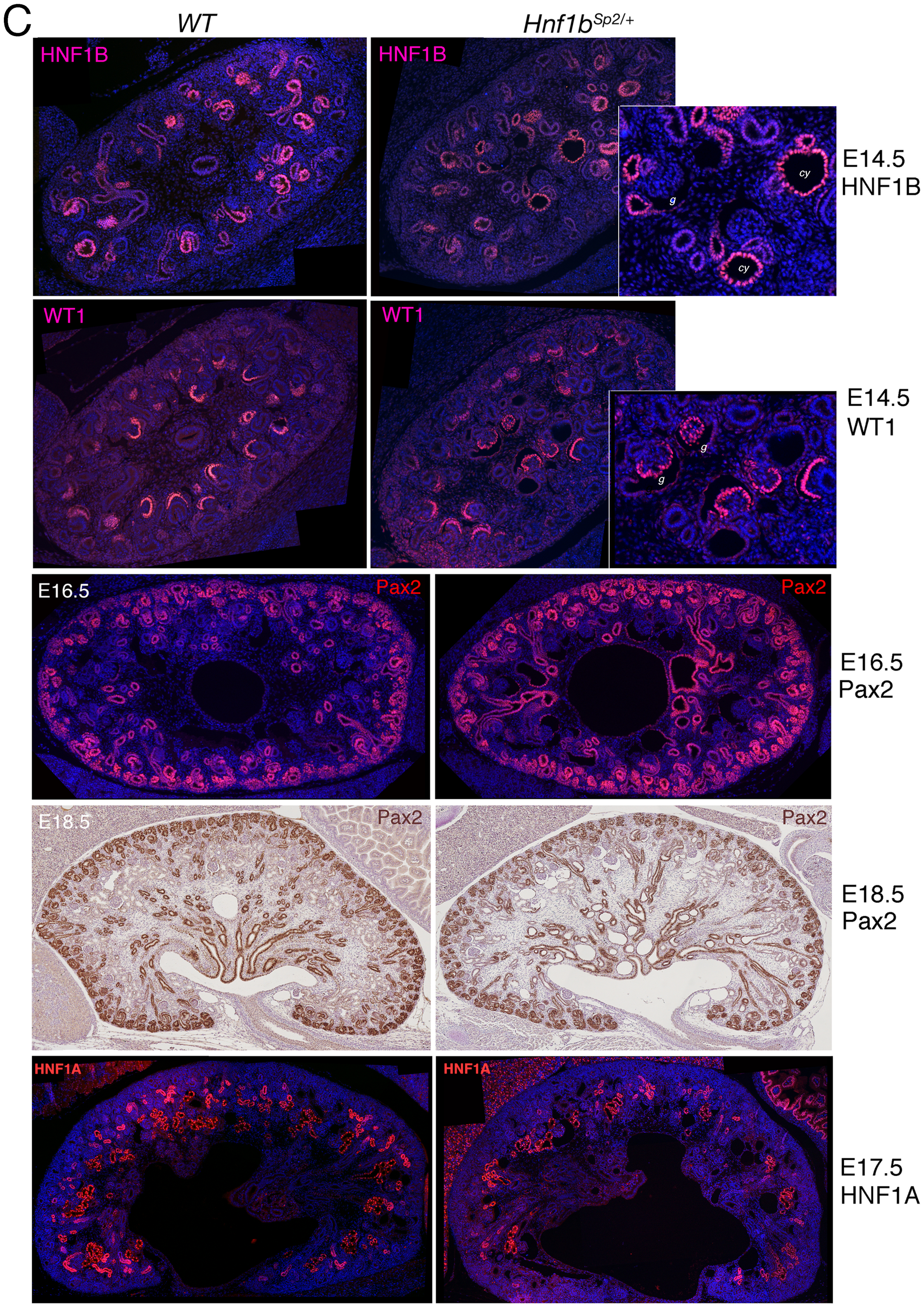

Figure S3C

Disease Models & Mechanisms • Supplementary information

Figure S4: **Representative Hematoxylin and Eosin (H&E) stained sections of P0 kidneys in the 129/sv and C57BL/6N background exhibiting increasing severity in the renal phenotype**. Note frequent pelvic dilatations and /or hydronephrosis in the C57BL/6N background. The last two C57BL/6N kidney sections correspond to the same *Hnf1b*$^{sp2/+}$ embryo indicating bilateral severely affected kidneys (duplication, hydronephrosis) that would likely be incompatible with life.

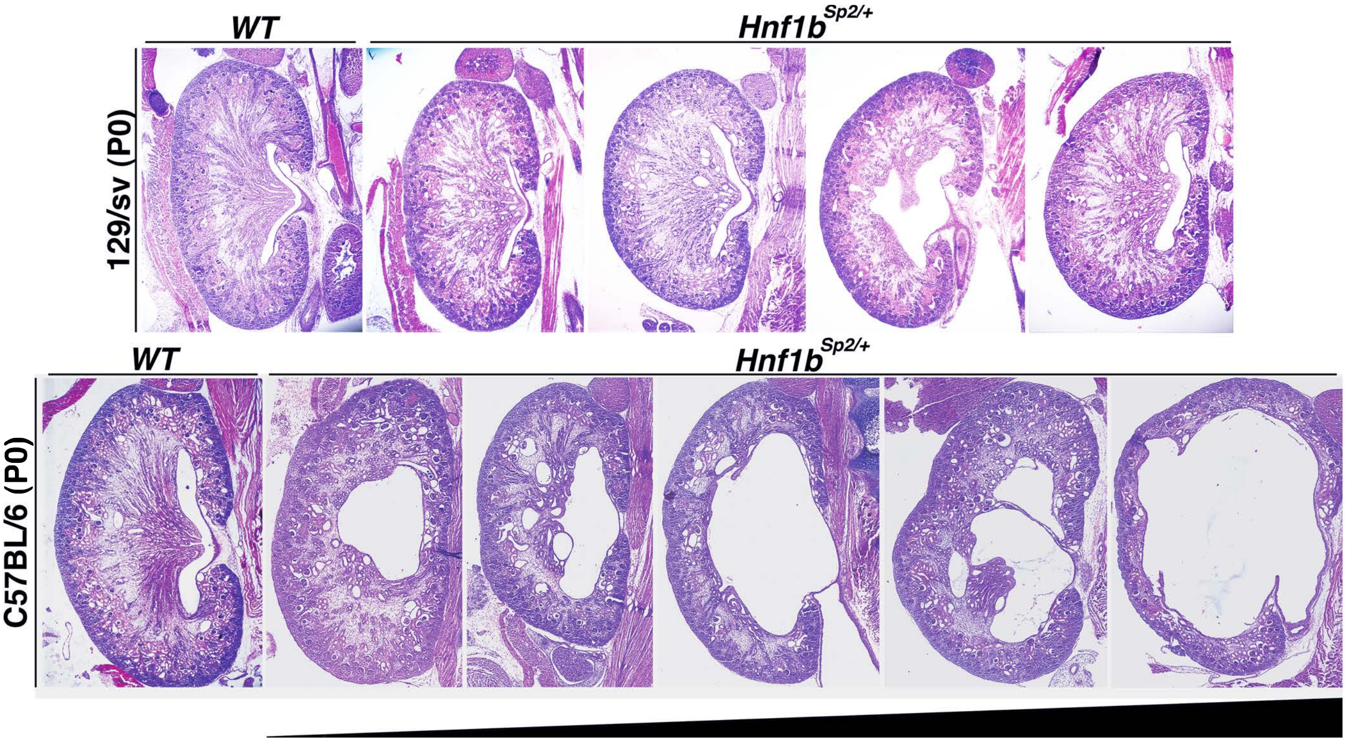

Figure S4

Disease Models & Mechanisms • Supplementary information

Figure S5: Immunohistochemical analysis of E17.5 and P0 kidney sections show defective proximal tubules cluster size and DBA collecting duct expression.

HNF4A and LTA staining show decreased and unequal clusters of PTs in *Hnf1b*$^{Sp2/+}$ kidneys at E17.5 (A’, B’) and P0 (F’, G’). NKCC2 a marker of the thick ascending Loop of Henle, stains predominantly medullar–cortical cystic structures (C’; H’), AQP2 and CK collecting duct markers express similar to *WT* (D, D’; I, I’; E, E’), while DBA is absent in *Hnf1b*$^{Sp2/+}$ collecting ducts P0 (J’). Sections were co-stained with DAPI. Scale bar: 200µm

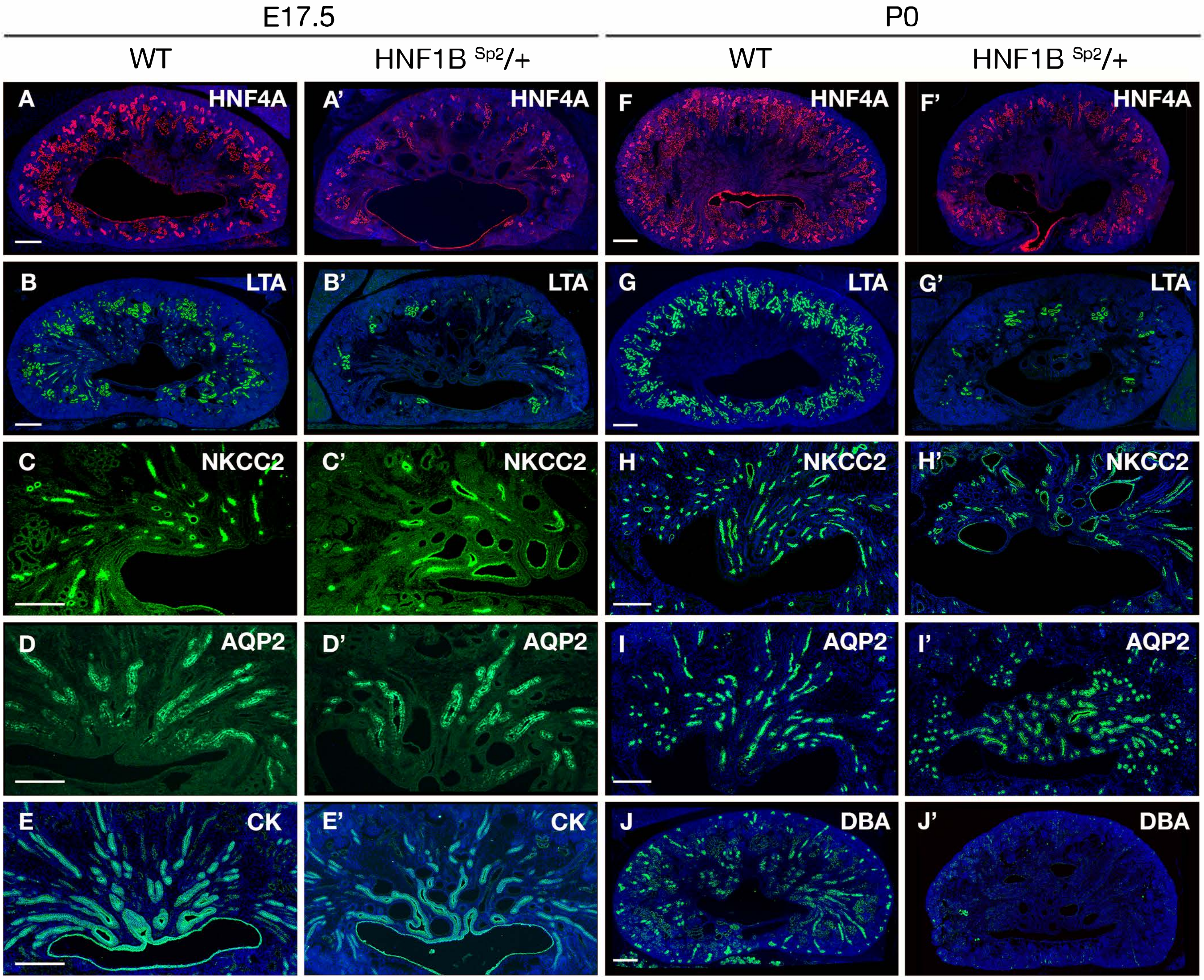

Figure S5

Disease Models & Mechanisms • Supplementary information

Figure S6: Increased proliferation in *Hnf1b*$^{Sp2/+}$ renal tubules

A) Representative images of phosphohistone H3 (H3P) and Pax2 co-stained E14.5 show similar numbers of H3P+ cells in epithelial tubular structures as compared with **WT as indicated the quantification shown on the right panel (***Hnf1b*$^{Sp2/+}$ n=3; WT n=3**). B**) Representative images of phosphohistone H3 (H3P) and HNF4A co-staining of E15.5 embryos show that dilated PTs exhibit an increased numbers of H3P+ cells per structure as compared with *WT*. **C**) Quantification of pH3-positive cells in PAX2 and H3P co-stained renal tubules of E16.5 *Hnf1b*$^{Sp2/+}$ embryos (n=8 sections; 3 embryos), indicates an increase of proliferating cells relative to *WT (*n=9 sections; 2 embryos*).* A higher and significant increase was also observed when compared proliferating cells of *Hnf1b*$^{Sp2/+}$ dilated/cystic tubules versus non-dilated tubules. Note also a significant reduction in the number of renal tubules of *Hnf1b*$^{sp2/+}$ relative to *WT*, while not significant (NS) changes in the number of nascent nephrons and glomeruli are observed. Unpaired *t*-test*.* p <0.01 (**) and p<0,0001 (***) are indicated

Disease Models & Mechanisms • Supplementary information

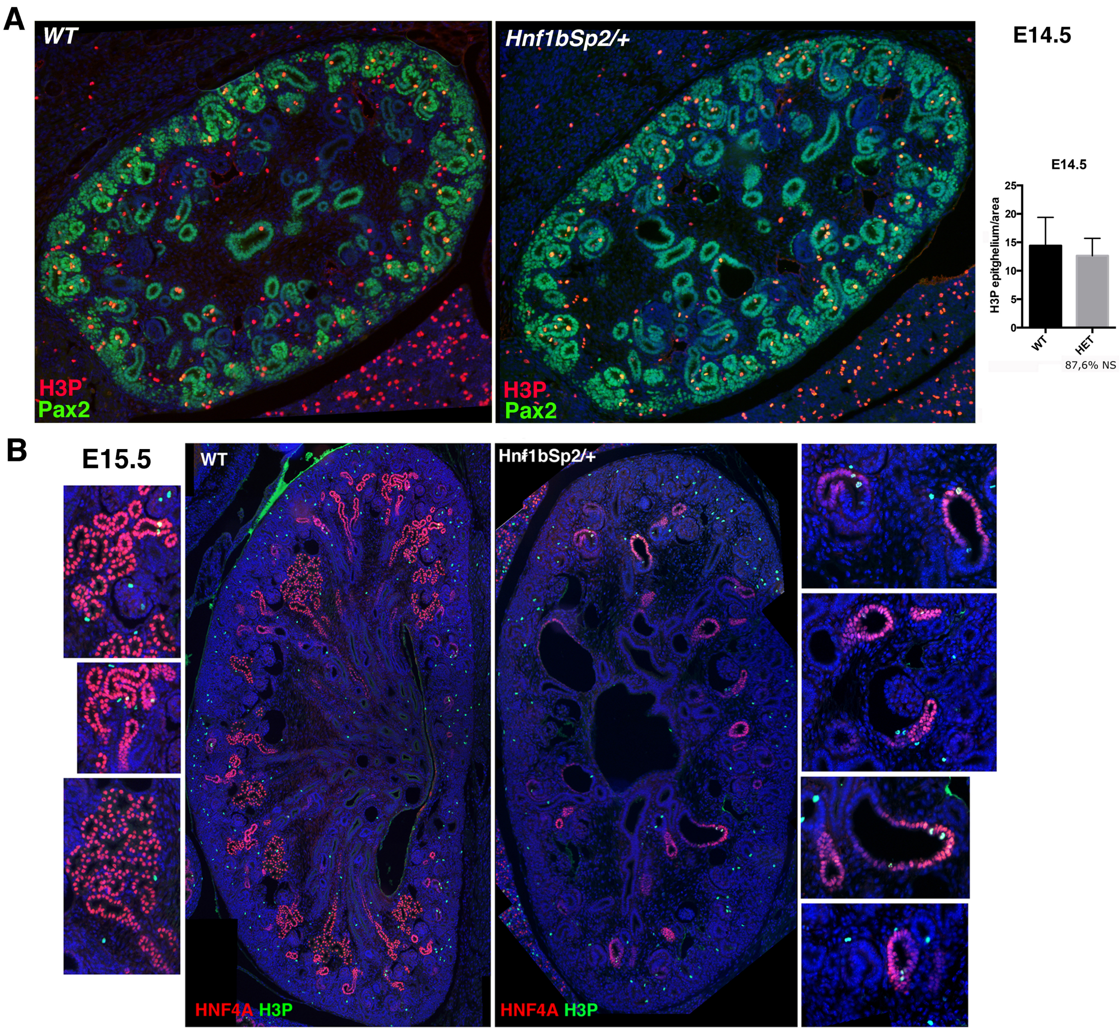

Fig.S6 A,B

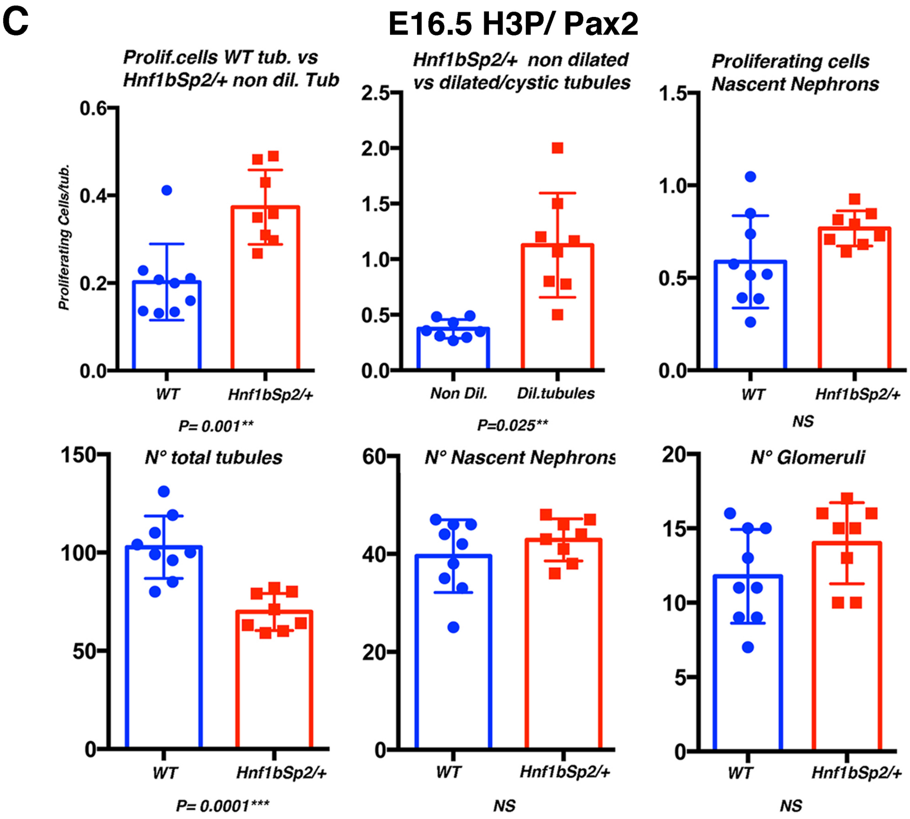

Figure S6C

Disease Models & Mechanisms • Supplementary information

Figure S7: HIS of early proximal tubule anchor genes *Fbp* and *Spp2*. mRNA levels of previously established *Hnf1b* target genes in *WT* and *Hnf1b*$^{Sp2/+}$ at different embryonic stages and at P0.

A: In situ hybridization at E17.5 and P0 of *WT* and *Hnf1b*$^{Sp2/+}$ kidneys show a strong decrease of *Fbp* at both stages and a more modest decrease of *Spp2*. **B**: mRNA levels of the indicated genes at E14.5, E15.5, E17.5 and P0 stages were determined by qRT-PCR and normalized by *cyclophilin-A* expression. Number of *WT* and *Hnf1b*$^{sp2/+}$ samples used are indicated, n being a pool of the 2 kidneys of each embryo. Values are represented as percentage of *WT* controls. Note the modest and non-significant downregulation of several target genes. Unpaired *t*-test. Significant differences between *WT* and *Hnf1b*$^{Sp2/+}$ (p<0.05 (*) and p <0.01 (**) are indicated. Error bars represent standard error of the mean (SEM).

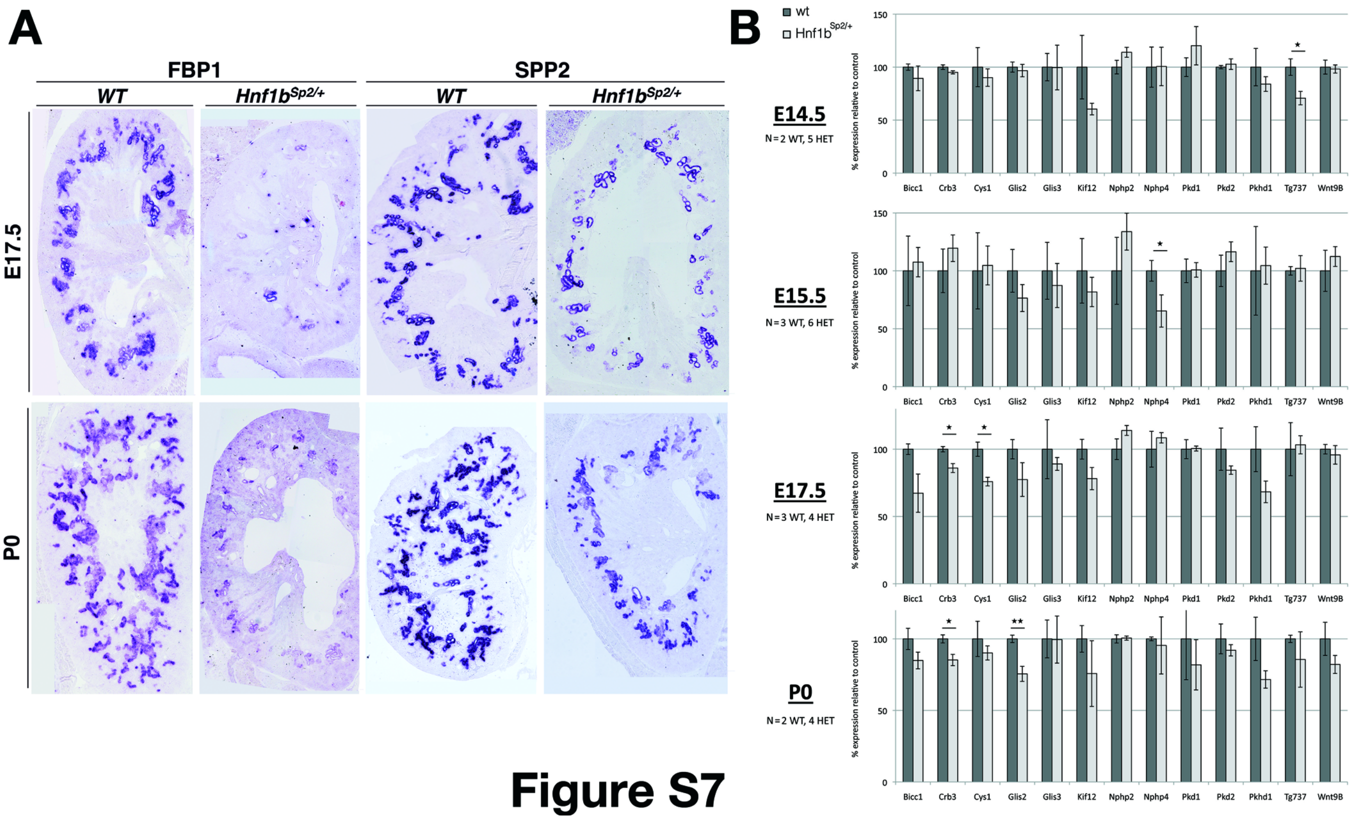

Figure S7

Disease Models & Mechanisms • Supplementary information

Figure S8: Comparison of different PT markers and previously identified *Hnf1b* target genes in E16.5 *Hnf1b*$^{sp2/+}$ versus *Hnf1a* null kidneys. Note that the PT markers HNF4A, CUBILIN, MEGALIN and LTA are not downregulated in *Hnf1a*$^{-/-}$ embryos (right panel), while are strongly downregulated in *Hnf1b*$^{sp2/+}$ embryos (middle panel) relative to *WT* (left panel) showing that during development the expression of PT genes does not depend on *Hnf1a*.

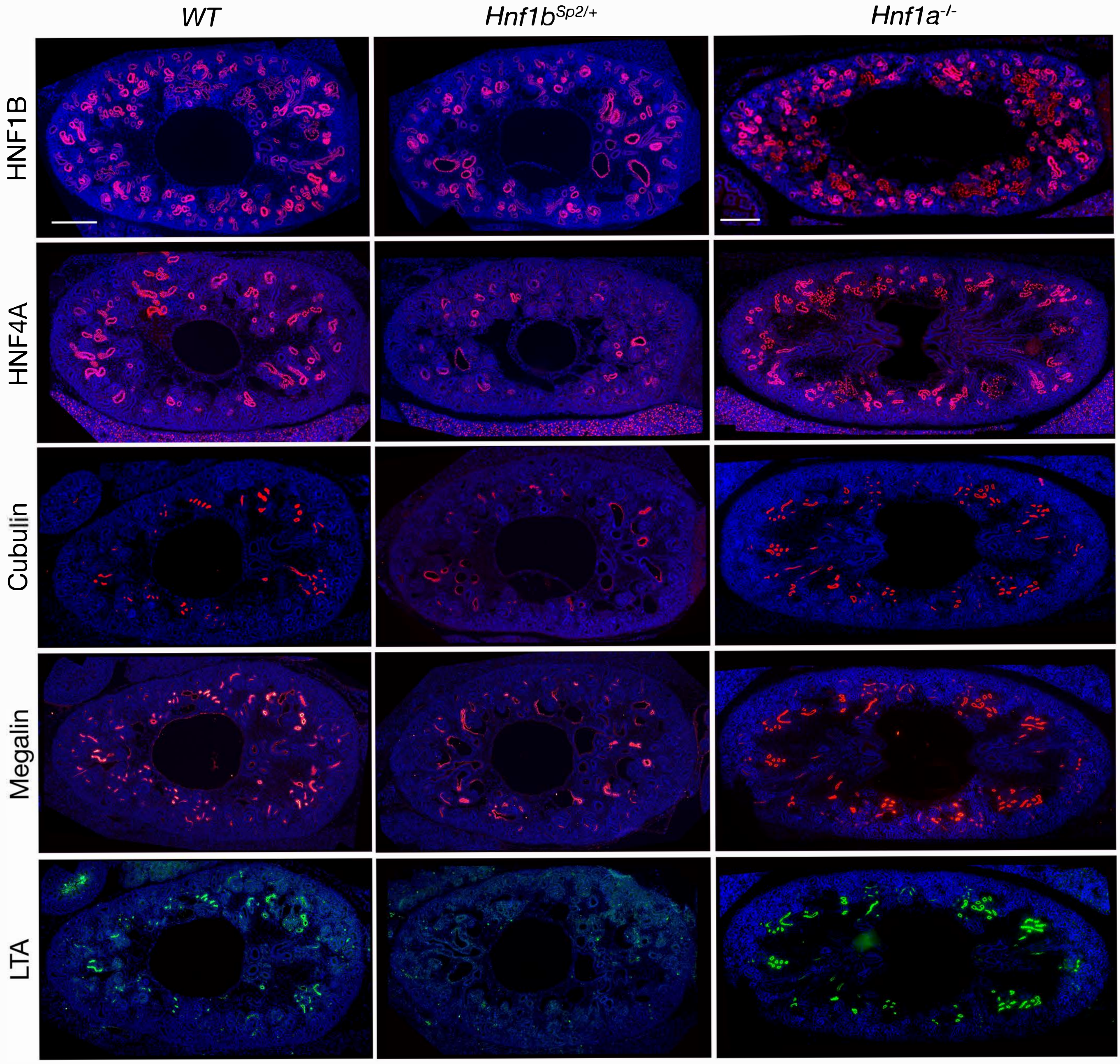

Figure S8

Disease Models & Mechanisms • Supplementary information

Figure S9: Representative histological sections of adult kidneys from *Hnf1*$^{Sp2/+}$ males and females illustrate disease variation. H&E stained sections of male kidneys of *WT* 12 months (A) and *Hnf1b*$^{Sp2/+}$ *(B- F)* showing kidney sections of 2month (B), 11 month- (C)' 5 month- (D), 17 month- (E), 12 month-old (F). H&E stained sections (G, J, K, L) and trichrome Masson (H, I) stained sections of female kidneys. G: *WT*; H to L of *Hnf1b*$^{Sp2/+}$ of 2-months (H), 12 months (I), 17 months (J), 15 months (K, L). Note clusters of glomerular cysts (magnifications shown in B', E', F', I', I"), microcysts (B, H, I), cell-debris within glomerular cysts (black arrow-heads and surrounding hemorrhagic regions (red arrow-heads), and severe hydronephrosis in both old males (E) and females (L) and rare cases of a severe dysplastic kidney (F). Note no evidence of fibrosis in trichrome Masson stained sections (H, I).

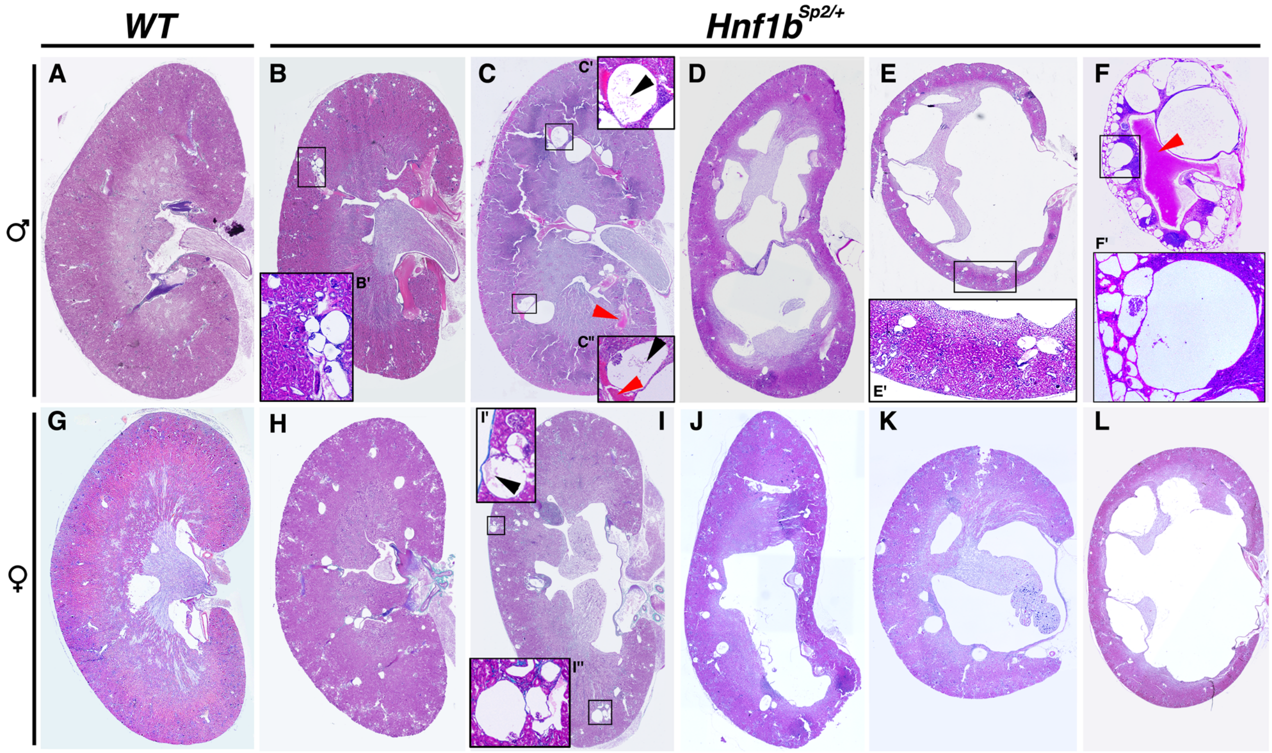

Figure S9

Disease Models & Mechanisms • Supplementary information

Figure S10: Representative histological sections of the Left and Right kidneys of *Hnf1b*$^{Sp2/+}$ adult males and females, illustrating the variable severity. Body weight curves and kidney weight/body weight ratios. H&E stained sections of the Left and Right kidneys of males (left panel) and females (right panel). **Male Age:** A, A': 17-months; B, B': 13-months; C, C': 10-months; D, D': 16-months; E, E': 9-months; F, F': 17-months; G, G': 17-months; H, H': 12 months. **Female Age:** A, A': 2 months; B, B': 13-months; C, C': 17-months. Unilateral hydronephrosis and pelvic dilatations in 6 to 17 months-old *Hnf1b*$^{sp2/+}$ mice were observed in respectively 41% and 14 % of *n*=43 males, and 33% and 27% of *n*=18 females. The right lower panel shows body weight curves of males (at the left) and females (at the right) at different ages. Note the tendency of 20% reduction in body weight of both *Hnf1b*$^{Sp2/+}$ males and females, although lower numbers of females were examined. Note also that one month-old heterozygous mutants exhibited lower kidney weight/body weight ratios than *WT* reflecting mild hypoplastic kidneys.

Disease Models & Mechanisms • Supplementary information

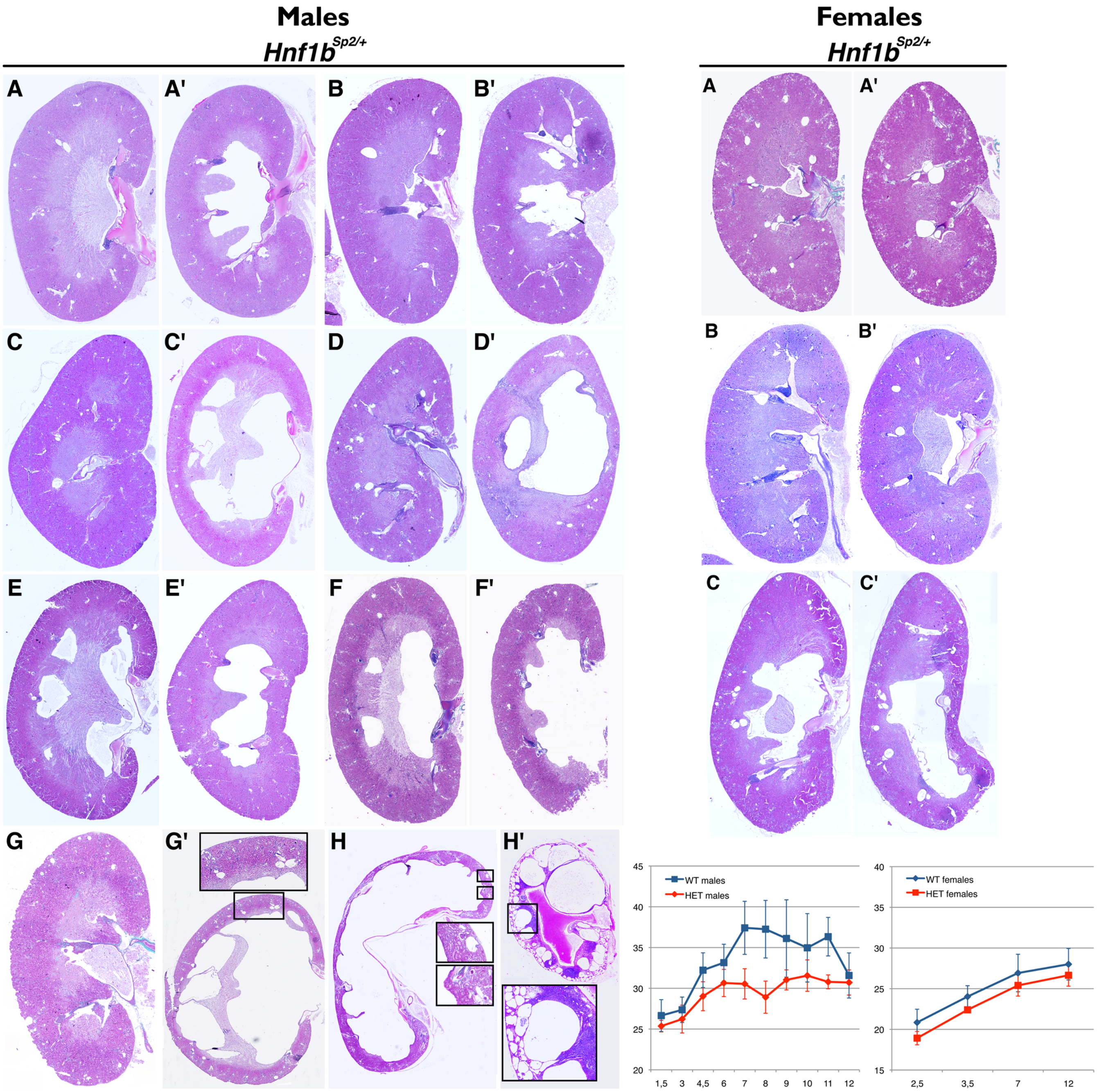

Figure S10

Disease Models & Mechanisms • Supplementary information

Figure S11: WT1 staining of E15.5 *Hnf1b*$^{Sp2/+}$ kidneys

E15.5 *Hnf1b*$^{Sp2/+}$ kidney section stained with WT1 showing a glomerular cyst with expanded Bowman's Capsula (BC) and glomerular tuft (gt) that have lost the expression of WT1. Note adjacent tubular cyst (cy) and normal WT1 expression of next podocyte layers, early nephron structures and condensed mesenchyme.

Hnf1b$^{Sp2/+}$

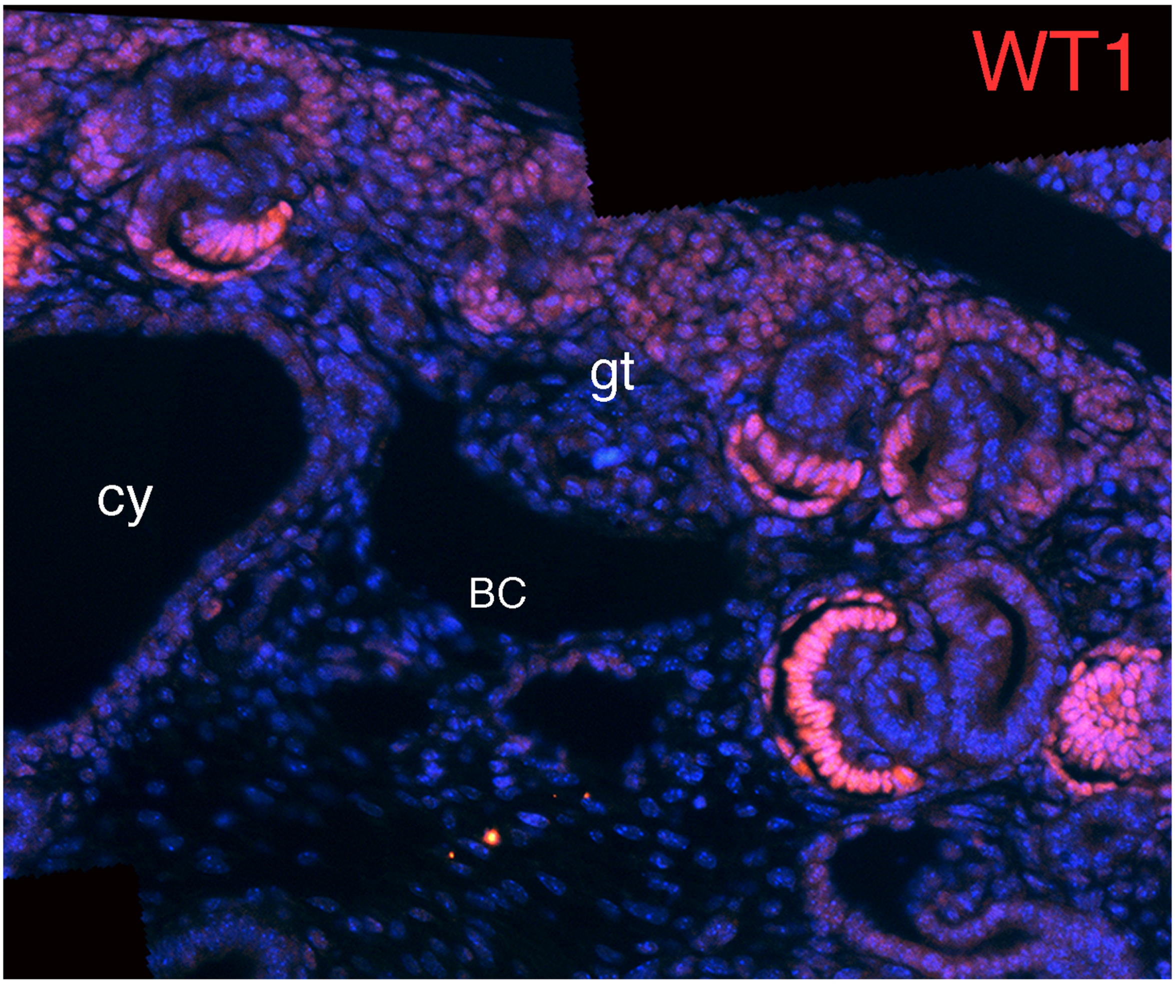

Figure S11

Disease Models & Mechanisms • Supplementary information

Table S1: Percentages of renal phenotypes at E18.5 -P0 and Mendelian inheritance

S1A: Percentages of renal phenotypes of *Hnf1b*$^{Sp2/+}$ at E18.5-P0

Hnf1b $^{Sp2/+}$ *Renal Phenotypes at E18.5-P0*	Fi	C57BL/6N	129/sv
N° kidney	28	14	10
Glomerular cysts (%)	82	83	70
Tubular cysts (PT & LOH) %	68	83	80
Pelvic Dilations (%)	3	41*	0
Hydronephrosis (%)		33	0
Duplicated kidney (%)	4	17	0
Bilateral (%)	86	93	80
Severity			
No phenotype (%)	14	0	20
Weak (%)	32	7	50
Moderate to severe (%)	32	43	30
Severe (%)	22	50	0

Phenotypes were defined on the basis of histological analyses of whole kidneys and do not take in account RNA/immunohistochemistry data analyses. WT embryos do not exhibit renal phenotype, but moderate pelvic dilations were observed in 5% of C57BL/N6 *WT* mice (*), suggesting that the *Hnf1b* splicing mutation exacerbates this phenotype. Note that in case of hydronephrosis, tubular dilations cannot be observed.

Table S1B: Mendelian inheritance in the offspring of *Hnf1b*$^{Sp2/+}$ and *WT* intercrosses in different mouse backgrounds

	C57BL/6N				129/Sv				F1			
**Stage**	***Hnf1b*$^{Sp2/+}$**	***WT***	**TOT**	**%HET**	***Hnf1b*$^{Sp2/+}$**	***WT***	**TOT**	**%HET**	***Hnf1b*$^{Sp2/+}$**	***WT***	**TOT**	**%HET**
**E14.5**	25	32	57	43,8					18	11	29	62
**E15.5**	13	15	28	46,4	9	9	18	50	9	10	19	47,3
**E17.5**	13	12	25	52	9	12	21	42,8	16	19	35	45,7
**P0**	10	12	22	45,4	10	8	18	55,5	20	21	41	48,7
**Adult**	(125*)	223	348	35,9	241	265	506	47,6	99	133	232	42,6
	(118*)	223	341	33,9								

Are indicated the number of embryos and/or animals of *WT* and heterozygous *Hnf1b*$^{sp2/+}$ mutant obtained from *Hnf1b*$^{Sp2/+}$ males x *WT* females in the indicated genetic backgrounds, the total embryos or animals (TOT) and percentages of heterozygotes. Numbers with * in C57BL/6N background mice indicate that 7 *Hnf1b*$^{Sp2/+}$ mice died from P1 and P25. The causes of death were not defined due to cannibalism.

Disease Models & Mechanisms • Supplementary information

Table S2: Primers used for Q- and semi-Q-RT-PCRs

Gene symbol	Gene name	RefSeq (NCBI)	Primers (5'-3')
Aqp2	aquaporin 2	NM_009699	CCATTGGTTTCTCTGTTACCCTG<br>CGGTGAAATAGATCCCAAGGAG
Bicc1	protein bicaudal C homolog 1 isoform 2	NM_001347189	TGAGGTCAGAACACCTACGA<br>AGTGCAGTGAAGACTGTCCA
Crb3	crumbs family member 3	NM_001347408	CCTGTCTTCAGGGGCTATTG<br>TCGCATGAGCAGAAACAGTC
Cyclo	cyclophilin A	NM_120034.2	CAGGTCCTGGCATCTTGTCC<br>TTGCTGGTCTTGCCATTCCT
Cys1	cystin 1	NM_001004455	AGAGGAGCTCATGGCGAGCATT<br>CTGTGGCACAGATGCCAAGAGG
Glis2	GLIS family zinc finger 2	NM_031184	GACGAGCCCCTCGACCTAA<br>AGCTCTCGATGCAAAGCATGA
Glis3	GLIS family zinc finger 3	NM_001305671	TGTGGCATGAATCTCCACCG<br>TGATGGGAGGGATATGTTGACC
Hnf1a	hepatocyte nuclear factor 1 (HNF-1), alpha	NM_009327.3	GTGTAACTGCACAGGAGGCAAA<br>TTCTCACGTGTCCCAAGACCTA
Hnf1b: WT transcript in Hnf1b$^{Sp2/+}$ samples	hepatocyte nuclear factor 1 (HNF-1), beta	NM_001291268.1	CGAGGTGGACCGGATGCTCA<br>TCGGAGGATCTCCCGTTGCT
Hnf4a	hepatocyte nuclear factor 4 (HNF-4), alpha	NM_001312906.1	CCATCATCTTCTTTGATCCAG<br>CTCACTTGCACCTGTGACC
Ihh	Indian hedgehog	NM_001313683	CCACGTGCATTGCTCTGTCA<br>CACGCTCCCCGTTCTCTAGG
Kcnj1	potassium inwardly-rectifying channel, subfamily J, member 1	NM_001168354	TGTCTCCACGTTTACCCCAGCA<br>CGTTGCCGAGAACGCCCAAATA
Kif12	kinesin family member 12	NM_001317352	GAGGCCAATAGCATCAACCG<br>GGAGATGCAGTGGCCCAAT
Lrp2	low density lipoprotein receptor-related protein 2	NM_001081088	AAAATGGAAACGGGGTGACTT<br>GGCTGCATACATTGGGTTTTCA
Nphp2	inversin	NM_010569	ACTTGTTACCCAGCATATGTGGTC<br>AGGAGAAAACATTTGAACCTTGTCTT
Nphp4	nephronophthisis 4 (juvenile) homolog (human)	NM_153424	TTGGCAATAAGCCAGAATCTCC<br>TGATACAGCCTCAACCGTTTGTC
Pkd1	polycystic kidney disease 1 homolog	NM_013630	GCTGCATGCCAGTTCTTTTG<br>TTTTAAAGTGCAGAAGCCCCA
Pkd2	polycystic kidney disease 2	NM_008861	CATGTCTCGATGTGCCAAAGA<br>ATGGAGAACATTATGGTGAAGCC
Pkhd1	polycystic kidney and hepatic disease 1	NM_153179	ATGGAGAACATTATGGTGAAGCC<br>ATGTTTCTGGTCAACAGCCC
Tg737	transgene insert site 737, insertional mutation, polycystic kidney disease	NM_009376	GCAGGCTTCAACCTCATCCTTA<br>TTTCTCCCGATCTCCAATGG
Tmem27	transmembrane protein 27	NM_001313719	TCTATCTGGAATCCGGCAAC<br>CACTCCAGGTGGTCCTTTGT
Wnt9B	wingless-type MMTV integration site family, member 9B	NM_011719	GTGAGAAGGAAGATGGTGAGC<br>GCAGAATCTGGAGAACTTGGC
Umod	uromodulin	NM_001278605.1	CTGCACCGATCCTAGTTCCG<br>TCTACCCTGCATTCTTCGCAA

Primers used for semiquantitative RT-PCR

Gene symbol	Gene name	RefSeq (NCBI)	Primers (5'-3')
vATG	hepatocyte nuclear factor 1 (HNF-1), beta	NM_001291268.1	TGGTGTCACAGCTCACGTC
V695	hepatocyte nuclear factor 1 (HNF-1), beta	NM_001291268.1	GAGCAGGTGTCCTCCGACTGC
Gapdh	glyceraldehyde-3-phosphate dehydrogenase	NM_001289726.1	TCCAGTATGACTCCACTCAC<br>ACCTTGCCCACAGCCTTG

Disease Models & Mechanisms • Supplementary information

Table S3: E14.5, E15.5, E17.5 and P1 transcriptomic profiles (Files 1-4). Shared differentially expressed genes between stages (File 5). Differentially expressed anchor early proximal tubules, proximal tubule, Loop of Henle/distal tubule at E17.5 and P1 (File 6). SLC-transmembrane transporters differentially expressed (File 7). Transcriptomic profile comparisons of *Hnf1b*$^{Sp2/+}$ at E17.5 and P1 and those reported for *Hnf4a* P1 mutant kidney (Marable et al., 2020) (File 8).

Click here to download Table S3

Table S4: Gene ontology enrichment analysis of downregulated genes at E14.5, E15.5, E17.5 and P1. Selected Gene ontology (GO) terms of differentially expressed genes at different stages (File 5).

GO term enrichment analysis using ToppGene informatics at different stages (Chen et al., 2009), showing the most important GO-terms at each stage.

Click here to download Table S4

Disease Models & Mechanisms • Supplementary information

Table S5. Renal phenotypes, Kidney-to-body weight ratios and basal parameters of WT and *Hnf1b*$^{Sp2/+}$ adult mice

A. Percentages of renal phenotypes in adult *Hnf1b*$^{Sp2/+}$ mice

	Hnf1b$^{Sp2/+}$	*Hnf1b*$^{Sp2/+}$
Age: 6-15 months	Males*	Females
Number of mice	48	18
Unilateral hydronephrosis (%)	41	33
Pelvic dilatations (%)	14	27
Glomerular cysts (%)	80	85
Microcysts (%)	25	15
Renal dysplasia/hypoplasia (%)	2	

* Absence of apparent histological phenotype in 8% of males. *WT*s do not show renal phenotypes, excepted of pelvic dilatations in 3% of C57BL/6N adult mice (males/females).

B. Kidney-to-body weight ratios of *WT* and *Hnf1b*$^{Sp2/+}$ males

Age	1 month		5 months		10 months		12 months		16-20 months	
Parameter	***WT***	***Hnf1b***$^{Sp2/+}$	***WT***	***Hnf1b***$^{Sp2/+}$	***WT***	***Hnf1b***$^{Sp2/+}$	***WT***	***Hnf1b***$^{Sp2/+}$	***WT***	***Hnf1b***$^{Sp2/+}$
Body Weight (BW) (g)	20,2 ± 2,6	18,28 ± 0,83	35,1 ± 1,52	29,3 ± 6,46	31,2 ± 2,7	30,1 ± 1,5	37,7 ± 4,38	29,2 ± 1,77	33,1 ± 5,5	30,6 ± 6,2
Left Kidney Weight (KW)	0,14 ± 0,01	0,11 ± 0,02*	0,2 ± 0,02	0,19 ± 0,03	0,22 ± 0,04	0,22 ± 0,04	0,29 ± 0,05	0,17 ± 0,09	0,25 ± 0,06	0,22 ± 0,07
Right Kidney Weight (KW)	0,14 ± 0,01	0,11 ± 0,02*	0,22 ± 0,01	0,23 ± 0,04	0,24 ± 0,04	0,21 ± 0,04	0,26 ± 0,02	0,19 ± 0,001	0,27 ± 0,08	0,22 ± 0,06
KW (left) /BW	0,71 ± 0,05	0,58 ± 0,07*	0,6 ± 0,09	0,66 ± 0,24	0,72 ± 0,07	0,74 ± 0,15	0,77 ± 0,08	0,59 ± 0,13	0,75 ± 0,07	0,72 ± 0,19
KW (right) /BW	0,69 ± 0,05	0,59 ± 0,05*	0,65 ± 0,07	0,79 ± 0,03	0,78 ± 0,11	0,70 ± 0,15	0,72 ± 0,08	0,66 ± 0,05	0,82 ± 0,15	0,72 ± 0,13
Ratio KW (left+right) /BW	1,40 ± 0,1	1,16 ± 0,11*	1,24 ± 0,17	1,45 ± 0,27	1,50 ± 0,15	1,44 ± 0,27	1,49 ± 0,14	1,15 ± 0,18	1,58 ± 0,19	1,44 ± 0,23
	n=3	n=5	n=2	n=2	n=6	n=5	n=3	n=2	n=5	n=11

Values represent the mean ± SEM of the indicated numbers (n) of male mice. Animals with the 2 kidneys to total body weight ratio lower than 1.4 are considered as low. *Hnf1b*$^{Sp2/+}$ kidneys of one-month mice exhibit a moderate hypoplasia manifested by the low kidney-to-body ratio. Note also that there is a bias in these ratios in cases of pelvic dilations of *Hnf1b*$^{sp2/+}$. Unpaired 2-tailed *t*-test (*) P <0,05

C. Basal parameters of *WT* and *Hnf1b*$^{Sp2/+}$ males

AGE	5-6 months		12 months	
Basal parameters	***WT (n=11)***	***Hnf1b***$^{Sp2/+}$ ***(n=13)***	***WT (n=11)***	***Hnf1b***$^{Sp2/+}$ ***(n=14)***
Body weight (BW, g)	31,137 ±5,622	29,683 ±3,5	36,5 ± 5,39	35,87 ± 4,9
Water intake (ml/day)	3,909±1,43	5,361±1,81*	2,703±1,196	3,409±0,64
Water intake (20g BW)	2,51±0,91	3,61±1,2*	1,47±0,65	1,9±0,35
Urine volume (ml/day)	0,79±1,43	1,615±1,187*	0,79±1,43	0,79±1,43
Urine volume day (20g BW)	0,507±0,9	1,088±0,79*	9±1,43	1±1,43
Aliments (g/day)	3,91±1,46	3,39±0,58	1,15±0,7	1,58±0,9
RATIO	***Hnf1b*** $^{sp2/+}$ ***/ WT***		***Hnf1b*** $^{sp2/+}$ ***/ WT***	
Hnf1b$^{Sp2/+}$ */ WT Urine vol 20g BW*	**2,14***		**1,354**	
Hnf1b$^{Sp2/+}$ */ WT Water vol 20g BW*	**1,43***		**1,45**	

Values are the mean ± SEM and are the average of measurements from 2 days of urine sample collection under basal conditions at the Indicated ages. Urine samples were from 3 different groups of mice at 5-6 &12 months. Unpaired 2- tailed *t*-test (*) P <0,05.

D. Water deprivation for 22h in 5-6 month WT and *Hnf1b* $^{Sp2/+}$males

5-6 month	**WT (n=3)**	***Hnf1b Sp2/+* (n=4)**
**Urine volume (mL)**		
Baseline	0,99 ± 0,34	0,81 ± 0,38
Water deprivation	0,28 ± 0,12	0,51 ± 0,33
**Urine osmolality (mOsm/kg H2O)**		
Baseline	1713 ± 358	2060 ± 793
Water deprivation	3794 ± 508**	2837 ± 468*

Values are the mean ± SEM after 22h of water deprivation. Upon water deprivation *Hnf1b*$^{Sp2+}$ mice are less efficient than WT in decreasing urine volume excretion and increasing urine osmolality. n= number of mice.

Disease Models & Mechanisms • Supplementary information

TABLE S6 : Differentially excreted urinary peptides between *Hnf1b*$^{Sp2/+}$ and *WT* mice.

Sequence	Protein name	Accession number	Start (aa position)	Stop	Adjusted p-value	Fold Change
**DOWN-REGULATED**						
WTDVGMSPRIESASLQGSDRVL	Pro-epidermal growth factor	P01132	620	641	0.0385	-6,27
GRMAHASmGNRPYGPNMANMPPQVGS	AT-rich interactive domain-containing protein 1A	A2BH40	857	882	0.0009	-5,02
PAAPGPAGSPANDNGNGNGNGNGNGNGGKGKPA	Striatin-interacting proteins 2	Q8C9H6	4	36	0.002	-4,95
LSSLKHPSNIAVDPIERL	Pro-epidermal growth factor	P01132	161	178	0.0028	-4,54
SSLKHPSNIAVDPIERLM	Pro-epidermal growth factor	P01132	162	179	0.0063	-3,7
ALDYDPVESKIYFAQTA	Pro-epidermal growth factor	P01132	522	538	0.0005	-3,7
SGNFIDQTRVLNLGPITR	Uromodulin	Q91X17	590	607	0.0385	-2,81
SSLKHPSNIAVDPIERL	Pro-epidermal growth factor	P01132	162	178	0.0114	-2,78
GAGLEQEAAAG	Heterogeneous nuclear ribonucleoprotein U	Q8VEK3	70	80	0.0494	-2,76
SINKELQNSIIDL	Kidney androgen-regulated prot.	P61110	26	38	0.0385	-2,36
SPRIESASLQGSDRVL	Pro-epidermal growth factor	P01132	626	641	0.0304	-2,18
LVSINKELQNSIIDLLNS	Kidney androgen-regulated prot.	P61110	24	41	0.0448	-2,16
PGApGAPGHPGPPGPV	Collagen alpha-1(III) chain	P08121	1039	1054	0.002	-1,9
GPpGPpGPpGPpG	Collagen alpha-1(XV) chain	O35206	704	716	0.0436	-1,71
SINKELQNSIID	Kidney androgen-regulated prot	P61110	26	37	0.0132	-1,58
DGQPGAKGEpGDTGVKGD	Collagen alpha-1(I) chain	P11087	809	826	0.0193	-1,54
SpGPDGKTGPpGP	Collagen alpha-1(I) chain	P11087	535	547	0.0117	-1,18
**UP-REGULATED**						
PGAKGEpGDTGVKGD	Collagen alpha-1(I) chain	P11087	812	826	0.0311	1,29
pGpPGpRGPQGPNGADGPQGp	Collagen alpha-1(XI) chain	Q61245	1219	1239	0.0145	1,38
KpGERGLpGEF	Collagen alpha-2(I) chain	Q01149	575	585	0.0466	1,43
GpGERGEHGPpGP	Collagen alpha-1(III) chain	P08121	795	807	0.0212	1,53
GIpGTGGpPGENGKpGEpGP	Collagen alpha-1(III) chain	P08121	641	660	0.0032	1,62
NIGFpGPKGPSGDpGKpGERGHpG	Collagen alpha-2(I) chain	Q01149	497	520	0.0311	1,68
GPpGPTGPAGDKGD	Collagen alpha-1(III) chain	P08121	617	630	0.0348	1,69
GLPGpAGPpGEAGKpGEQ	Collagen alpha-1(I) chain	P11087	633	650	0.0009	1,69
GQPGAKGEpGDTGVKGDAGPpGP	Collagen alpha-1(I) chain	P11087	810	832	0.0348	1,74
DGTpGGpGIRGMpG	Collagen alpha-1(III) chain	P08121	526	539	0.0464	1,8
TGPIGpPGPAGApGDKGEA	Collagen alpha-1(I) chain	P11087	755	773	0.0287	1,87
GPpGEAGKpGEQ	Collagen alpha-1(I) chain	P11087	639	650	0.0375	1,94
GPIGPpGPAGQpGDKGEGGSpGLpG	Collagen alpha-1(III) chain	P08121	764	788	0.0132	2
EAGKpGEQGVpGDLGApGP	Collagen alpha-1(I) chain	P11087	643	661	0.0348	2,04
GQpGAKGEpGDTGVKGDAGppGP	Collagen alpha-1(I) chain	P11087	810	832	0.0348	2,16
RDGApGAKGDRGETGP	Collagen alpha-1(I) chain	P11087	1015	1030	0.0032	2,26
GLpGPAGPpGEAGKpGEQGVpG	Collagen alpha-1(I) chain	P11087	633	654	0.0231	2,31
TTGEVGKpGERGLpGEF	Collagen alpha-2(I) chain	Q01149	569	585	0.0375	2,48
GEpGAKGERGApGEKGEG	Collagen alpha-1(III) chain	P08121	818	835	0.002	2,72
AGQpGEKGPpGAQGPpGSpGPLG	Collagen alpha-1(III) chain	P08121	925	947	0.0054	3,06
EVGKpGERGLpGEF	Collagen alpha-2(I) chain	Q01149	572	585	0.002	3,13
pGPAGpPGEAGKPGEQGVpGDLG	Collagen alpha-1(I) chain	P11087	635	657	0.0005	6,9
GpPGEAGKpGEQGVpGDLGApGP	Collagen alpha-1(I) chain	P11087	639	661	0.0008	7,07

Data were obtained from 17 *Hnf1b*$^{Sp2/+}$ and 18 *WT* mice from 3 to 15 months. 40 peptides with different abundance in urine between the two groups were selected after multiple testing (**Material and Methods).** No differences were observed among different ages. *P* values were defined using Wilcoxon rank-sum test followed by adjustment for multiple testing. Peptides are separated according to *Hnf1b*$^{Sp2/+}$ mice urinary results (up- and down-excreted).

Disease Models & Mechanisms • Supplementary information

Table S7: List of antibodies

Primary Antibodies	Species	Source	Dilution
HNF1A	rabbit	M. DeVas and J. Ferrer (Imperial College London)	1/100
HNF1B	rabbit	Santa Cruz Biotechnology (H-85 sc22840)	1/100
HNF1B	rabbit	homemade (westerns)	1/500
HNF1B	rabbit	Sigma-Aldrich (HPA002083)	1/500
HNF4a	rabbit	Abcam (ab181604)	1/50
PAX2	rabbit	Covance (PRB-276P)	1/300
AQP1	rabbit	Alpha Diagnostic int. (AQP1 1-A)	1/300
AQP2	rabbit	M. Knepper NIH Bethesda	1/300
NKCC2	rabbit	M. Knepper NIH Bethesda	1/100
Uromucoid (Umod)	goat	MP Biomedicals, Illkrich, France (54140)	1/300
Collectrin	sheep	RD Systems (Ref AF4965)	1/250
LTA-biotin and FITC		Vector lab (FL-1321)	1/200
DBA Biotin and FITC		Vector lab (B-1035)	1/100
WT1	rabbit	Santa Cruz Biotechnology (C19-SC192)	1/200
Laminin	rabbit	Sigma-Aldrich; St. Louis, MO (L9393)	1/100
Calbindin D-28K	rabbit	Chemicon International (AB1778)	1/100
Pan cytokeratin	mouse	Sigma-Aldrich; St. Louis, MO (P2871)	1/50
E-cadherin	mouse	Sigma-Aldrich; St. Louis, MO (610182)	1/200
MUC1	armenian hamster	Neomarkers; Fremont, CA (Muc1 Ab-5)	1/100
Acetylated Alpha - Tubulin	mouse	Sigma-Aldrich; St. Louis, MO (T6793)	1/100
ZO-1	rabbit	Zymed laboratories (40-2300)	1/100
Vil1	rabbit	Sylvie Robine, Institut Curie, France	1/200
CUBN	rabbit	Renata Kozyraki (Cordeliers Research Center)	1/1000
LRP2	sheep	Renata Kozyraki (Cordeliers Research Center)	1/1000
Phospho Histone H3	mouse	Cell Signaling (6G3)	1/200
Alpha-Tubulin	monoclonal	Sigma-Aldrich; St. Louis, MO (T9026)	1/10.000
**Secondary Antibodies**	**species**	**Source**	**Dilution**
Alexa Fluor 488	rabbit	Invitrogen	1/500
Alexa Fluor 488	mouse	Invitrogen	1/500
FITC Armenian hamster	Armenian hamster	Jackson (127-095-160)	1/500
Biotin rabbit		Vector	1/1000
Biotin mouse		Vector	1/1000
Biotin Goat		Vector	1/1000
Streptavidin-Alexa 594		Jackson ImmunoResearch	1/500
Streptavidin-Alexa 488		Jackson ImmunoResearch	1/500
Peroxidase (westerns)	Rabbit	Jackson ImmunoResearch	1/10.000
Peroxidase (westerns)	Mouse	Jackson ImmunoResearch	1/10.000

Disease Models & Mechanisms • Supplementary information

Table S8: Summary of RNA samples from embryonic kidneys at the indicated stages employed in RNA-seq analysis

Stage	*WT*	*WT*	***Hnf1b***$^{Sp2/+}$	***Hnf1b***$^{Sp2/+}$
**E14.5**	SCR10-E145-4wt	SCR13-E144wt	SCR12-E145 HET	SCR15-E145-HET
**E15.5**	*WT* (Pool *n*=3)		TR (Pool *n*=3)	
**E17.5 ***	GZS 17	GZS 35 (*n*=3)	GSZ 34 (*n*=3)	GSZ 36 (*n*=3)
**P1**	SCR20_P1_	SCR21_P1_	SCR22 P1	SCR23_P1

*RNA was prepared from the 2 kidneys of each embryo, except when the n is indicated (*i.e. *E15.5 and E17.5) * RNA from 2 litters born the same day in the same cage (Pool n=3, 6 kidneys)*

Disease Models & Mechanisms • Supplementary information
